# Tissue and time specific expression pattern of interferon regulated genes in the chicken

**DOI:** 10.1186/s12864-017-3641-6

**Published:** 2017-03-28

**Authors:** Susanne Röll, Stefan Härtle, Thomas Lütteke, Bernd Kaspers, Sonja Härtle

**Affiliations:** 1 Department for Veterinary Science, University of Munich, Munich, Germany; 2 formerly Department for Veterinary Science, University of Munich, Munich, Germany; 3 Institute of Veterinary Physiology and Biochemistry, JLU Giessen, Giessen, Germany

**Keywords:** Chicken, IFNα, IRG, Half-life, Expression profile

## Abstract

**Background:**

Type I interferons are major players against viral infections and mediate their function by the induction of Interferon regulated genes (IRGs). Recently, it became obvious that these cytokines have a multitude of additional functions. Due to the unique features of the chickens’ immune system, available data from mouse models are not easily transferable; hence we performed an extensive analysis of chicken IRGs.

**Results:**

A broad database search for homologues to described mammalian IRGs (common IRGs, cIRGs) was combined with a transcriptome analysis of spleen and lung at different time points after application of IFNα. To apply physiological amounts of IFN, half-life of IFN in the chicken was determined. Interestingly, the calculated 36 min are considerably shorter than the ones obtained for human and mouse. Microarray analysis revealed many additional IRGs (newly identified IRGs; nIRGs) and network analysis for selected IRGs showed a broad interaction of nIRGs among each other and with cIRGs. We found that IRGs exhibit a highly tissue and time specific expression pattern as expression quality and quantity differed strongly between spleen and lung and over time. While in the spleen for many affected genes changes in RNA abundance peaked already after 3 h, an increasing or plateau-like regulation after 3, 6 and 9 h was observed in the lung.

**Conclusions:**

The induction or suppression of IRGs in chickens is both tissue and time specific and beside known antiviral mechanisms type I IFN induces many additional cellular functions. We confirmed many known IRGs and established a multitude of so far undescribed ones, thus providing a large database for future research on antiviral mechanisms and additional IFN functions in non-mammalian species.

**Electronic supplementary material:**

The online version of this article (doi:10.1186/s12864-017-3641-6) contains supplementary material, which is available to authorized users.

## Background

Interferons (IFNs) are cytokines critically involved in the control of viral, bacterial, fungal and parasitic infections. They mediate their activity either directly or indirectly through the induction of regulators of innate and adaptive immune cells [1]. Three families of IFNs have been described in mammals. In humans type I IFNs form a multigene family with 13 members of closely related *IFNα* genes, a single distinct *IFNβ* gene. These cytokines are best known for their antiviral activity and were the first IFNs identified [2, 3]. In addition, there are genes for *IFNε*, *IFNκ*, and *IFNω*, whose function is less well characterized [1]. Type II IFN is represented by a single gene encoding IFNγ. IFNγ has pleiotropic effects on cells of the immune system and is known as a potent activator of macrophages [4]. More recently, a third IFN family was uncovered named type III IFN which in humans comprises four *IFNλ* genes. IFNλs exert similar responses as type I IFNs but their activity is largely restricted to epithelial tissues as a consequence of the restricted expression of IFNλ receptors [5]. Type I IFNs are induced in response to viral infections in most cell types. Viral infections are sensed by the cells through pattern recognition receptors (PRRs) located in the cytoplasm or the endosomal compartment. RIG-I and MDA-5 are the primary but not only cytosolic sensors recognizing RNA. The endosomal PRRs (TLR3, TLR7/8) are double and single stranded RNA sensors of the Toll-like receptor (TLR) family. In contrast, TLR9 binds unmethylated CpG DNA. Upon ligand binding these PRRs activate downstream signals such as IRF3 and IRF7 which induce IFN gene transcription and secretion [6, 7].

Type I IFNs bind to a common receptor (interferon-α/β receptor (IFNAR)) expressed on most cell types. Ligation of the heterodimeric receptor activates the JAK/STAT signaling pathway which leads to phosphorylation of STAT1 and STAT2 and together with IRF9 to the formation of the ISGF3 complex which induces transcription of IFN regulated genes (IRGs) through binding to IRG response elements [8]. Several IRGs have been studied in great detail including myxovirus resistance 1 (MX1), IFN-inducible double-stranded RNA-dependent protein kinase (PKR), 2’-‘5-oligoadenylate synthetase (OAS) and IFN induced transmembrane proteins (IFITMs) [9]. Besides this canonical pathway, type I IFNs induce the formation of STAT1 homodimers and the induction of γ-activated sequences (GAS) in promoter regions typically activated by IFNγ. Furthermore, signaling through STATs 3, 4 and 5 has been described. While signaling through the STAT1/STAT2 pathway induces classical antiviral responses, alternative signaling leads to the production of a broad range of cytokines, chemokines, antimicrobial products and regulators of apoptotic pathways [10]. Transcriptomic studies have shown the induction of hundreds of genes upon type I IFN receptor ligation in vitro and in vivo [11–13]. Recently, based on these studies large scale screening systems were established to identify IRGs that are induced by different viruses to elucidate their functional relevance [10, 14].

While in-depth knowledge on the mammalian IFN system has been accumulated over decades as outlined, comparatively little is known in avian species. This is surprising taken the fact that poultry flocks are worldwide under intensive pressure by viral infections, which lead to huge economic losses. Most recent examples are highly pathogenic avian influenza (HPAI) outbreaks 2014/2015 in North America with estimated 50 million birds dead or culled [15] or the constant threat by velogenic Newcastle Disease outbreaks around the globe [16].

Members of all three IFN families have been identified in different avian species such as chickens, turkeys and ducks. In chickens the type I family is smaller than in humans with approximately ten IFNα genes and a single IFNβ gene [17, 18]. Cloning and expression of recombinant proteins enabled functional studies and confirmed the antiviral properties of avian type I IFNs in vitro and in vivo as well as the induction of known IRGs including Mx, OAS and PKR.

Though in general the mammalian and avian IFN systems appear quite similar, when studied in more detail subtle differences became apparent between birds and mammals and between individual bird species. In an effort to get insights into the differences in HPAV infection outcomes between chicken and ducks, it was recognized that galliformes lack the PRR RIG-I which strongly induces type I IFN production in mice. As this molecule is present in the duck genome and fully functional, this lead to the conclusion that observed differences in HPAIV susceptibility might result from insufficient activation of the type I IFN pathway in chickens [19]. However, work in chickens infected with the HPAIV H5N1 demonstrated rapid induction of IFNα transcripts and protein indicating that alternative pathways such as the MDA-5 and/or the TLR pathways may account for the response [20]. Interestingly, such a loss in the family of RIG-I like receptors was not only found in birds but also in mammals as recently reported for RIG-I in the Chinese tree shrew [21].

To a limited extent IRGs have been investigated in more detail in birds [22–24]. In analogy to the mammalian system the avian Mx orthologue was considered to have potent antiviral properties [25, 26]. Surprisingly, in vitro and in vivo studies clearly demonstrated that chicken Mx does not limit influenza virus replication despite strong induction of the protein by IFN [27, 28]. Thus, it remains to be elucidated which IRGs confer antiviral resistance in different viral disease. Such knowledge is crucial to form breeding programs for increased innate resistance and to understand the unique antiviral properties amongst avian species.

To lay the foundation for future research we treated chickens with recombinant IFNα at physiological plasma levels and performed intensive gene expression analysis in lung and spleen. Here we provide a comprehensive list of chicken IRGs and show that in addition to common IRGs shared between mammals and chickens unique chicken IRGs could be identified. This information together with recently published in vitro studies will allow large scale functional screens of IRGs as recently reported in mammals [29].

## Methods

### Animals

Fertilized eggs of conventional White Leghorn chickens (LSL) were obtained from Lohmann Tierzucht GmbH, Cuxhaven, Germany and hatched at the Institute of Animal Physiology, Munich. Birds were vaccinated against Marek’s Disease Virus after hatch, housed under conventional conditions in aviaries with groups up to ten birds and received food and water ad libitum.

### Interferon treatment of chickens

Recombinant chicken interferon alpha (rec chIFNα) was expressed and purified from Escherichia coli as described previously [30]. The concentration of chIFNα was determined using an internal standard initially qualified with the international standard 67/18 (80 IU/ml) [31] and reported in Units per ml (U/ml). For injection, appropriate amounts of rec chIFNα for pharmacokinetics and the microarray study were dissolved in PBS (pH 7,2).

### Pharmacokinetics

Six weeks old LSL chickens received a single i.v. injection of 1×10^7^ Units for the biggest bird and a direct proportional weight adjusted dose for all others. Plasma samples were collected 5, 10, 15, 30, 60, 120, 240, 360 and 480 min after dosing and chIFNα levels were determined. Pharmacokinetic parameters were calculated by Non Compartmental Analysis using Microsoft Excel® as follows: The highest plasma concentration observed (Cmax) as well as the corresponding time point (Tmax) were directly derived from the time/concentration plot for each individual animal. ChIFNα plasma Clearance (Cl) was calculated as a direct function of dose and Area Under the time/concentration Curve (AUC) (i.e. Cl = Dose/AUC). AUC was determined using the linear-trapezoidal rule (i.e. sum of the areas below the time/concentration curve with a linear connection between consecutive data points). The volume of distribution (Vz) as well as the terminal elimination half-life (T1/2) were extrapolated based on an exponential regression of the last three time/concentration points.

### Experimental design of the microarray experiment

Twenty-four six-week-old LSL chickens were divided into four groups, a control group and three groups which received 1x10^7^U rec chIFNα into the V. jugularis or V. cutenea ulnaris superficialis every 3 h. Birds were sacrificed at three hours (one IFN application), six hours (two IFN applications) and nine hours (three IFN applications) after the first treatment. The control group received a single injection of buffer and was killed after 6 h. Blood samples of each animal were taken before injection and before killing and obtained plasma was stored at -80 °C. Spleen and lung samples were isolated and stored in RNA later (Ambion, Warrington, UK) at -80 °C until RNA isolation. Lung samples were taken by selecting the second to last segment of the left lung containing the primary and secondary bronchi of the torus intercostalis.

### IFN assay

Type I IFN activity in plasma samples was determined using a bioassay based on the strong induction of the *Mx* gene in the presence of type I IFN as described recently [30]. Briefly, CEC-32 reporter cells containing a luciferase gene under the chicken Mx promoter were incubated with serial dilutions of plasma samples or the chIFNα standard. After 6 h of incubation cells were lysed with 100 μl of 1× cell culture lysis reagent (Promega, Madison, USA) and luciferase activity was measured using Luciferase substrate (Promega, Madison,USA) and a Glomax 96 microplate luminometer (Promega, Madison, USA).

### IL6 assay

The amount of IL6 protein in plasma samples of rec chIFNα treated chicken was measured by the ability of added plasma to stimulate proliferation of the IL6-dependent murine hybridoma cell line 7TD1 (kindly provided by J. Van Snick, Ludwig Institute for Cancer Research, Brussels, Belgium) essentially as described previously [32]. Briefly, 7TD1 cells were thoroughly washed in PBS and cultured with 5×10^3^ cells per well of a 96-well flat-bottomed microtiter plate in medium (RPMI 1640 medium with Glutamax, 10% fetal bovine serum) and serial twofold dilutions of the samples or of 1 ng/ml *E.coli* derived recombinant chIL-6. Cells were cultured for 4 days and proliferation was measured using the XTT [2,3-bis(2-methoxy-4-nitro-5-sulfophenyl)-5-((phenylamino)carbonyl)-2H-tetrazolium hydroxide] colorimetric assay [33]. XTT (Sigma, Taufkirchen, Germany) was diluted in RPMI 1640 medium to a final concentration of 1 mg/ml and phenazine methosulfate was added to a final concentration of 0.025 mmol. Fifty microliters of the XTT/phenazine methosulfate solution were added to each well, and the cells were incubated at 37 °C and 5% CO_2_ for 4 h before absorbance was measured in a photometer at 450 nm.

### RNA isolation

Lung and spleen tissue was homogenized in peqGold TriFast (Peqlab, Erlangen, Germany). Lung tissue was homogenized with a rod homogenizer (Ultra-Turrax, IKA, Staufen, Germany). Spleen tissue was homogenized with a tissue homogenizer (Precellys 24, Peqlab, Erlangen, Germany). Total RNA was isolated according to the manufacturer’s Trizol protocol. Quantity and purity of extracted RNA was determined with a NanoDrop 1000 (PEQLAB Biotechnologie GMBH, Erlangen, Germany), while the RNA quality was analysed using a 2100 Bioanalyzer® (Agilent Technologies, Böblingen, Germany). Only RNA samples with an RNA integrity number (RIN) exceeding 8.4 were used for qRT-PCR and microarray analysis.

### Oligonucleotide microarray analysis

Microarray analysis was performed using Agilent 4×44K chicken-genome microarrays, supplemented by the addition of 1699 selected genes out of the chicken genome, supposed to play a crucial role in the chicken immune system (AMADID 23824). The best four out of six lung and spleen samples of each group in the IFN experiment were hybridised. After hybridising, washing and scanning of the microarrays image following manufacturer’s instructions (Agilent technologies, Santa Clara, US), arrays were further processed with Feature Extraction Software 10.5.1.1 (Agilent Technologies). Processed signals were filtered based on “Well above background” flags (detection in three of four samples in either one of the experimental groups) and normalized using the BioConductor package vsn [34]. The quality of the normalized data was checked with a distance matrix and a heatmap following on pair-wise distances (BioConductor package geneplotter). Significance analysis was performed with the Microsoft Excel add-in “Significance analysis of microarrays” (SAM; multiclass) [35]. With a False discovery rate (FDR) <5% a fold change (FC) higher than 2 and smaller than -2 was accepted as significant.

The original Agilent gene annotation was refined by BLAST search in NCBI and Ensembl and by AvI-Blast, a software especially designed to align oligonucleotide sequences to the chicken genome database of NCBI. Official gene symbols were obtained by online ID-converter DAVID, Babelomics and g:Profiler. Multi experiment viewer (MeV) was used for the generation of expression profiles, PANTHER gene list analysis to map genes to different gene ontology clusters. The INTERFEROME and ISG databases [11, 12, 36–39] were used as sources for known mammalian IRGs.

### Quantitative Real-Time RT-PCR

The same RNA samples as for the microarrays were used for quantitative real-time RT-PCR. Genomic DNA was eliminated by DNase I (Fermentas, St. Leon-Rot, Germany) and 400 ng cDNA were generated using the GOScript Reverse Transcription System (Promega Corporation, Madison, USA) according to the manufacturer’s instructions. 10 ng cDNA of spleen and lung were analysed for the relative abundance of 18S, *Mx, IL6* and *PKR* RNA with a GoTaq qPCR Master Mix (Promega Corporation, Madison, USA). Primers for qRT-PCR were designed using PerlPrimer software and obtained from Eurofins MWG Operon [40]. The following forward and reverse primers were used for qRT-PCR reactions: 18S rRNA: forward primer 5′-CATGTCTAAGTACACACGGGCGGTA-3′ and reverse primer 5-′GGCGCTCGTCGGCATGTATTA-3′ *IL6* forward primer 5′-GCTTCGACGAGGAGAAATGC-3′and reverse primer 5′-GCCAGGTGCTTTGTGCTGTA-3′ *Mx* forward primer 5′-GCGGACAAGCCATAGAACAAG-3′ and reverse primer 5′-GGCACCCCAAAAACTCCTACA-3′ *PKR* f+orward primer 5′-GGAAGTAGACATTTATGCGCTGG -3′and reverse primer 5′-CATCCTGCCATACCTTGTTTTTCT-3′. Quantitative RT-PCR was performed using a 7300 Real-Time PCR System® (Applied Biosystems, Warrington, UK) with SYBR-green. Obtained CT values were normalized to 18S rRNA (= dCT) and fold changes were calculated in comparison to the control group (2^-ΔΔCT^ method).

### Promotor-analysis

Sequences of differentially expressed and unaffected genes were analysed for the existence of Interferon stimulated response elements (ISRE) and/or Gamma activated sequence (GAS) elements in a first step by using ISRE and GAS sequences earlier described by Tsukahara et al. [41], who characterized functional ISRE and GAS elements according to a particular algorithm (Additional file 1: Table S1). As REFINEMENT, the search framework specifically designed for an automated large scale search for ISRE and/or GAS motifs isn’t available anymore, we screened chicken genes with motifs, which were described by Tsukahara et al. as “functional motifs”. Based on the work of Schuhmacher et. al. [22] the sequence 6 kb upstream of the first exon was considered as promotor region. In addition, the complete gene sequence was analysed from 6 kb upstream, including all exons and introns. Screening of Ensembl based sequences was performed with a self-designed Shell script. For a more detailed analysis the 30 genes with the highest changes in mRNA abundance after IFN injection were individually searched for the existence of the common ISRE consensus 5′A/GGTTTCN_(1-2)_TTTCC/T3′ or its reverse complement and the common GAS consensus 5′TTNCNNNAA3′ [42–45].

## Results

### Identification of IRGs by database analysis

In order to identify IRGs in the chicken we started with an extensive comparative database analysis to relate known mammalian IRGs to annotated chicken genes. Therefore entries in INTERFEROME, the ISG-database, KEGG [46], Reactome and several publications were combined and compared to all annotated 13,353 genes on a customized laboratory internal Agilent 4x44K chicken Genome microarray. Thereby we were able to identify 1420 genes, which are known as mammalian IRGs and are also present in the chicken genome. These genes were termed “common IRGs” (cIRGs) (see Additional file 2: Table S2 for Gene description, Accession numbers and sequences). Using the INTERFEROME database the identified cISGs were also assigned to type I, type II and type III interferons as inducers. From the identified 1420 ISGs, with 1192 the majority of genes are known to be induced by type I interferon, 898 by type II and only 86 by type III, though many IRGs are induced by several types of IFN leading to an extensive overlap.

### Pharmacokinetics of rec chIFNα

**Fig. 1:**
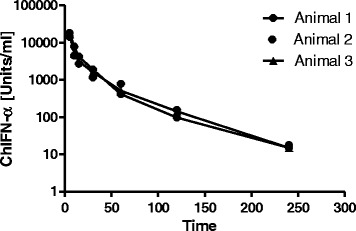
Pharmacokinetic profile of recombinant ChIFNα after i.v. injection. Three chickens were treated i.v. with a single dose of recChIFNα (1x10^7^Units for the biggest bird and a direct proportional weight adjusted dose for the others). Plasma was taken at the indicated time points, and the amount of biological active type I IFN was determined using an IFN reportergene assay

Next, we wanted to confirm the *in silico* identified cIRGs in vivo by treating chickens with recombinant chIFNα. In order to obtain biological relevant plasma levels of type I IFN for a longer time we first determined the pharmacokinetic properties of rec chIFNα in the chicken. Therefore, three adult chickens received a single i.v. injection of rec chIFNα. Plasma samples were collected at frequent intervals post dose and levels of chIFNα were determined using a reporter-assay for chicken type I IFN. Based on these results C_max_ at 16,624 ± 1,985 U/ml was achieved as early as five minutes post application (i.e. the first sampling time point post administration). The pharmacokinetic profile was characterized by a 2-compartment profile consisting of an initial distribution phase up to 60 min post injection and followed by 2^nd^ phase characterized by a first order elimination. At 240 min post application the lower detection limit of the bioanalytical assay was reached. (Fig. 1). From these data a mean terminal half-live of chIFNα was calculated at 36 min (SD 2.53 min, CV 7%). A detailed overview on all pharmacokinetic parameters calculated is provided in Additional file 3: Table S3.

### Microarray based identification of chicken ISGs

#### Experimental setup

**Fig. 2:**
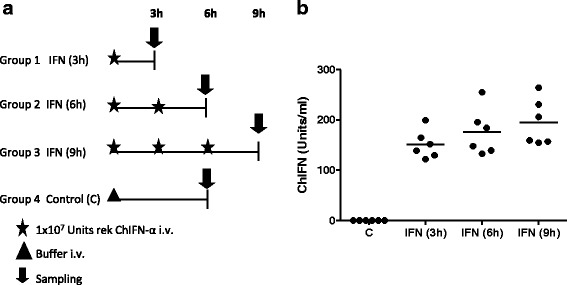
IFN treatment for the microarray experiment. **a** Four groups of chickens (*n* = 6 per group) received recChIFNα as indicated in the experimental scheme. **b** At the indicated time points animals were sacrificed and the plasma concentration of biologically active type I IFN was measured using an IFN reporter gene assay. Shown are group mean and individual IFN concentrations

In order to evaluate chicken ISGs on the transcriptome level rec chIFNα was applied to three groups of six week old chickens according to the experimental scheme shown in Fig. 2a. It is known from infection studies with a Newcastle disease virus (NDV) vaccine or Highly pathogenic Avian influenza viruses (HPAIV) that endogenous plasma levels of type I IFN range between 100 U/ml (NDV, own unpublished observations) and 1,000 U/ml (HPAIV, [20]). Therefore, based on the calculated half-life birds received an i.v. injection of 1×10^7^ U chIFNα every 3h to obtain a constant biologically relevant chIFN plasma level. After three (IFN(3 h)), six (IFN(6 h)) and nine hours (IFN(9 h)), respectively, a group of birds was killed and tissues were taken for RNA isolation and microarray analysis. Lung parenchyme (without BALT) was chosen as mucosal tissue serving as portal of entry for many pathogens, while the spleen represents the most prominent lymphatic tissue in the chicken. The control group received only buffer and was killed after 6 h.

To verify the treatment schedule plasma samples were obtained before the first IFN injection and before tissue sampling and analysed for the amount of chIFNα. While no type I IFN was detectable in any of the samples before treatment (data not shown) and in the control group, IFN treated groups had mean IFN plasma concentrations of 150 U/ml after three, 175 U/ml after six and 200 U/ml after 9 h (see. Fig. 2b). This demonstrates that the applied treatment schedule indeed led to a relatively constant IFN exposure of the animals.

### Identification of differentially abundant genes

**Fig. 3:**
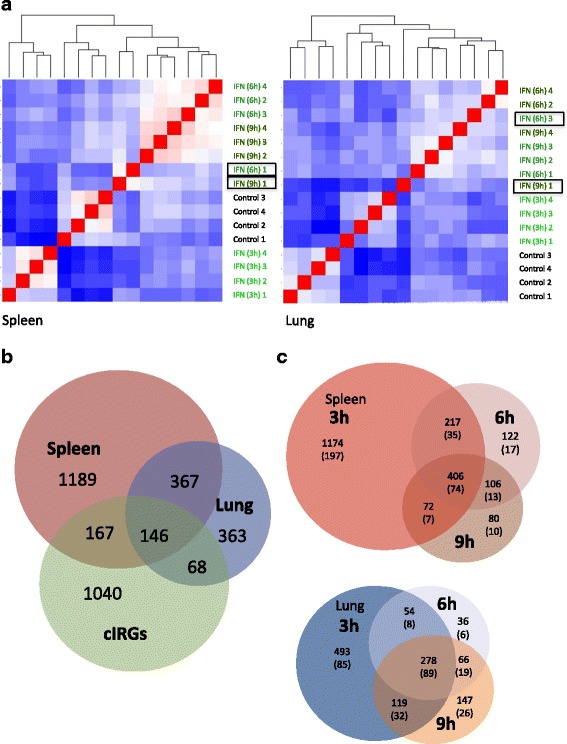
Microarray analysis after IFN injection. **a** “R” based heat map of spleen and lung tissue: Probes are labeled by group membership and animal number (1-4). Data sets with an unusual assignment which deviates from the rest of the group are *boxed*. **b** Venn diagram for the comparison of microarray and *in silico* analysis: Shown are the intersections of significantly regulated genes 3 h after IFN injection between spleen (*red*), lung (*blue*) tissue and the “common IRGs” (*green*), identified by database analysis. **c** Intersections of IRGs in spleen and lung at different time points after IFN injection: *Numbers* indicate array-based significantly regulated genes; *numbers in brackets* represent “common IRGs”

RNA from spleen and lung samples was hybridized to the customized Agilent 4x44K chicken array. Heatmap analysis using “R” demonstrated that IFN treatment resulted in significant transcriptome alterations and a distinct clustering of the different experimental groups (Fig. 3a). In both tissues all control and IFN(3 h) samples were clearly assigned to single groups, while for IFN(6 h) and IFN(9 h) in spleen and lung one sample did not group with the others. Interestingly, in the spleen, IFN-induced transcriptome changes in the three hours group were so pronounced, that the transcriptome after 6 and 9 h was closer related to controls than to this group. In the lung, control samples show a distinct separation from all IFN treated groups. Among the later IFN(3 h) is clearly discriminable from the other time points, while the transcriptome differences between 6 and 9 h were less pronounced and some overlap could be observed.

This observation is also reflected by the number of significantly regulated genes, that is genes with an FC ≤ -2 or ≥ 2 and an FDR ≤ 0.05. With 1870 (spleen) and 945 (lung), by far most regulated genes were identified in the three hours group (Table 1). It is noticeable that in the spleen at each time point down-regulated genes outnumber up-regulated ones, while in the lung three to fivefold more up-regulated genes were identified.

**Table 1:** Number of differentially expressed genes after IFN injection with a FC ≤ -2 or ≥ 2

		# of genes		
Spleen	IFN (3 h)	1869	+	871
			-	998
	IFN (6 h)	852	+	316
			-	536
	IFN (9 h)	665	+	257
			-	408
Lung	IFN (3 h)	945	+	685
			-	260
	IFN (6 h)	435	+	372
			-	63
	IFN (9 h)	611	+	489
			-	122

A clear overlap exists between the genes regulated after 3 h in lung and spleen, but many genes are also tissue specific (Fig. 3b). Five hundred thirteen genes were identified in both tissues representing about a quarter of all genes regulated in the spleen and more than half of the genes, which were regulated in the lung. When the genes were compared with the identified cIRGs, some intersections were found, but in total only a quarter (381 genes, representing 17% of splenic genes and 23% of lung genes) of the *in silico* identified cIRGs were also found in the in vivo experiment. Forty-six additonal cIRGs were found among the genes exclusively regulated after IFN(6 h) and IFN(9 h).

A comparison between the different time points revealed, that more than 60 and 50% of genes in the spleen and lung, respectively, were solely regulated in the three hours group. Seventy percent of splenic and 77% of lung genes regulated after 6 h showed also an altered abundance after 3 h. From the genes with altered abundance after 9 h almost 90% of genes in the spleen and 75% of genes in the lung were also found at earlier time points (Fig. 3c). The percentage of cIRGs among these gene subgroups was largely the same, ranging between 9 and 15% in the spleen and 13 and 23% in the lung.

Collectively, the observed variations in RNA abundances were much stronger in the spleen than in the lung. Fold changes for significantly regulated genes ranged from a 685fold up-regulation of *PTX3* (pentraxin related gene, rapidly induced by IL1beta) to a 436fold down-regulation of *SFTPA1* (surfactant pulmonary-associated protein A1) in the spleen (Table 2) and a 118fold up-regulation of *TULP1* (tubby like protein 1) to a 18fold down-regulation of *IRG1* (immunoresponsive 1 homolog) in the lung (Table 2). Most of the highest fold changes in both organs were found after 3 h, though for some of the 30 genes with highest changes in RNA abundance, e.g. *SFTPA1* the highest value was achieved after 9 h of IFN exposition (Table 2). Also among these 30 genes were the classical IRGs *Mx1* and Oligoadenylate-Synthase (*OAS*). With 36% (spleen) and 43% (lung) the percentage of cIRGs among these genes is twofold higher than among the entity of differentially expressed genes. The fact that only 30% of the splenic genes with the strongest regulation show also regulated expression in the lung and 23% of the lung genes are regulated in the spleen, demonstrate an distinct response to IFN stimulation in both organs.

**Table 2:** Top 30 of genes with the highest or least changes in mRNA abundance in spleen (A) and lung (B). The scanned region for promotor elements ranged from 6 kb upstream to 1 kb downstream of the first exon

Gene symbol	Gene description	Maximal FC	Timepoint of maximal FC	cIRG	Expression in lung	# of Promotor elements (ISRE/GAS)
A)						
PTX3	Pentraxin related gene, rapidly induced by IL1 beta	685	3 h	x	x	0/7
SFTPA1	Surfactant, pulmonary-associated protein A1	−436	9 h	x	-	0/12
LOC396260	Mature avidin	384	9 h	-	x	1/7
IL6	Interleukin-6	186	3 h	x	x	0/8
LAO	L-amino-acid oxidase precursor (Interleukin-4 induced 1)	169	3 h	-	x	0/7
K203	Chicken chemokine (C-C motif) L i 3	145	3 h	-	x	0/6
LL	Lung lectin	−140	9 h	-	-	0/12
PSCA	Prostate stem cell antigen	−124	9 h	-	-	0/12
KCNA1	Potassium voltage-gated channel, shaker-related subfamily, member 1	−69	3 h	-	-	0/5
LYG2	Lysozyme G-like 2	62	3 h	-	x	0/6
CSF3	Colony stimulating factor 3	58	3 h	x	x	0/3
NEFH	Neurofilament, heavy polypeptide	−56	3 h	-	-	0/5
CMBL	Carboxymethylenebutenolidase homolog	44	3 h	-	x	0/12
GCH1	GTP cyclohydrolase 1	43	3 h	x	x	0/9
FSHR	Follicle stimulating hormone receptor	−39	3 h	-	-	0/5
ZPD	Zona pellucida protein D	38	3 h	-	-	0/13
IRG1	Immunoresponsive 1 homolog	35	3 h	-	x	0/9
BATF3	Basic leucine zipper transcription factor, ATF-like 3	35	3 h	-	x	0/11
IL22	Interleukin-22	34	3 h	x	x	0/4
MAOA	Monoamine oxidase A	33	9 h	-	x	0/11
HPS5	Hermansky Pudlak syndrome 5	30	6 h	x	x	0/10
ETV1	ETS variant gene 1	−30	3 h	-	-	0/13
TULP1	Tubby like protein 1	28	3 h	-	x	0/11
MMP1	Matrix metallopeptidase 1 (interstitial collagenase)	27	3 h	-	x	1/7
LIPI	Lipase, member I	26	3 h	-	x	1/9
RPL37	Ribosomal protein L37	26	3 h	x	x	0/9
GBP	Guanylate binding protein	25	3 h	x	x	0/5
OAS	2‘5’oligoadenylate synthetase like	25	3 h	x	x	0/0
SPINK5	Serine peptidase inhibitor, Kazal type 5	−25	6 h	-	-	0/7
Mx1	Myxovirus (influenza virus) resistance-1	24	3 h	x	x	1/16
B)						
TULP1	Tubby like protein 1	118	3 h	-	x	0/11
IL6	Interleukin-6	67	9 h	x	x	0/8
CMBL	Carboxymethylenebutenolidase homolog	53	3 h	-	x	0/12
LOC396260	Mature avidin	44	9 h	-	x	1/7
LAO	L-amino-acid oxidase precursor	40	3 h	-	x	0/7
MSC	Musculin (activated B-cell factor-1)	33	3 h	-	x	0/5
FKBP5	FK506 binding protein 5	30	3 h	x	x	0/6
TGM4	Transglutaminase 4 (prostate)	24	3 h	-	-	0/10
LYG2	Lysozyme G-like 2	24	3 h	-	x	0/6
BATF3	Basic leucine zipper transcription factor, ATF-like 3	22	3 h	-	x	0/11
GBP	Guanylate binding protein	22	3 h	x	x	0/5
RPL37	Ribosomal protein L37	20	3 + 9 h	x	x	0/9
IFIT-5	Interferon induced protein with tetratricopeptide repeats 5	18	3 + 9 h	x	x	1/9
IRG1	Immunoresponsive 1 homolog	19	9 h	-	x	0/9
TLR5	Toll-like receptor-5	−18	3 h	x	-	0/11
SEMA6D		−17	3 h	-	x	0/11
GCH1	GTP cyclohydrolase 1	17	3 h	x	x	0/9
GAL9	Gallicin 9	16	3 h	-	-	0/8
Rd	Riboflavin-binding protein	16	3 h	-	-	0/15
MAOA	Monoamine oxidase A	16	9 h	-	x	0/11
SAMD9L	Sterile alpha motif domain containing 9 like	15	3 + 9 h	x	x	0/13
K203	Chicken chemokine (C-C motif) L i 3	14	9 h	-	x	0/6
IL1RL1	Interleukin-1 receptor like 1	14	3 h	x	x	0/15
MX1	Myxovirus (influenza virus) resistance-1	13	3 + 6 + 9 h	x	x	1/16
SOCS3	Suppressor of cytokine signaling 3	12	9 h	x	x	1/9
RASD1	RAS, dexamethasone induced 1	12	3 h	-	x	0/3
OAS	2‘5’oligoadenylate synthetase like protein	11	3 h	x	x	0/0
ALB	Albumin	10	3 h	-	-	0/12
PDK4	Pyruvate dehydrogenase kinase, isozyme 4	10	3 h	x	x	0/12
SNAP25	Synaptosomal-associated protein, 25 kDa	−10	3 h	x	-	0/19

Interestingly, with a 384fold and 44fold (spleen) and a 186fold and 67fold up-regulation (lung), mature avidin (LOC396260) and *IL6* (interleukin 6) were in both organs among the five strongest regulated genes. It is also noticeable that in the spleen several of the top 30 genes were cytokines and chemokines (*IL6, K203, CSF3, IL22*).

The vast majority of transcriptome changes occurred 3 h after IFN aplication and for genes, which were solely regulated at later time points, the regulation due to secondary effects becomes more likely. Hence, we decided to use only those genes which were regulated after 3 h for further analysis. Regulated genes which were not identified as cIRGs were termed as “newly identified IRGs” (nIRGs).

### Promotor analysis

Interferons function by receptor binding, activation of the JAK-STAT-pathway and finally binding of transcription factors to DNA regulatory elements in upstream regions of target genes: these are the so called interferon-stimulated response elements (ISREs) and gamma-activated sequences (GASs). In order to test whether the identified nIRGs could be activated by either of these response elements, we searched for ISREs and GASs in the respective genes.

Using an automated search we first analysed cIRGs, nIRGs and control genes, whose RNA abundance was neither in the spleen nor in the lung affected by IFN treatment for the presence of known ISRE and GAS (Table 3). When a region 6 kb upstream of the first exon to the last exon was taken as a basis for the search, ISRE and GAS were identified in about a quarter of the genes with a somewhat higher frequency (25 and 26%) in cIRGs and nIRGs than in control genes (22%) and the majority of regulatory elements being GAS. Interestingly, when only the region 6 kb upstream of the transcription start site, presumably the promotor region, was taken into account the percentage of genes containing ISRE and/or GAS motifs was in general very low and only slightly higher in cIRGs (6%) and nIRGs (5%) than in control genes (4%).

**Table 3:** Number and portion of chicken genes containing ISRE and/or GAS motifs

	Input	Localisation	ISRE/GAS	Percent
Not sign. after IFN	9677	whole gene	87/2117	22
		6 kb upstream	10/406	4
cIRG	383	whole gene	3/94	25
		6 kb upstream	3/20	6
nIRG	2254	whole gene	13/575	26
		6 kb upstream	3/102	5

Regions analysed were either 6 kb upstream of the first exon of the respective gene referred to as “6 kb upstream” or from 6 kb upstream of the first exon down to the last exon of the gene sequence, referred to as “whole gene”

Tsukahara et al. had also found that in a standard search, genes with higher mRNA abundance after IFN treatment, genes with lower mRNA abundance and randomly expressed genes contained almost the same portion of ISRE and GAS elements [41]. To overcome this problem, we performed a detailed manual search for the 30 most strongly affected genes (Additional file 4: Table S4). With the exception of OAS, where neither an ISRE nor a GAS was found, all top 30 genes contained several GAS elements. ISRE were only found in four genes in the spleen (avidin, *MMP1*, *LIPI, Mx*) and three genes in the lung (*IFIT5, Mx, SOCS3*) with avidin, *MMP1* and *LIPI* being nIRGs the others cIRGs. As Tsukahara et al. also proposed an accumulation of ISRE close to the transcription start site, we confined our search to 500 bp upstream of the transcription start site. However, in this region we found only one ISRE for *IFIT5* and a strongly reduced number of GASs.

In summary, ISREs and GASs could be identified only in a minority of regulated genes but with identical results for cIRGS and nIRGs.

### IRGs exhibit different expression profiles

**Fig. 4:**
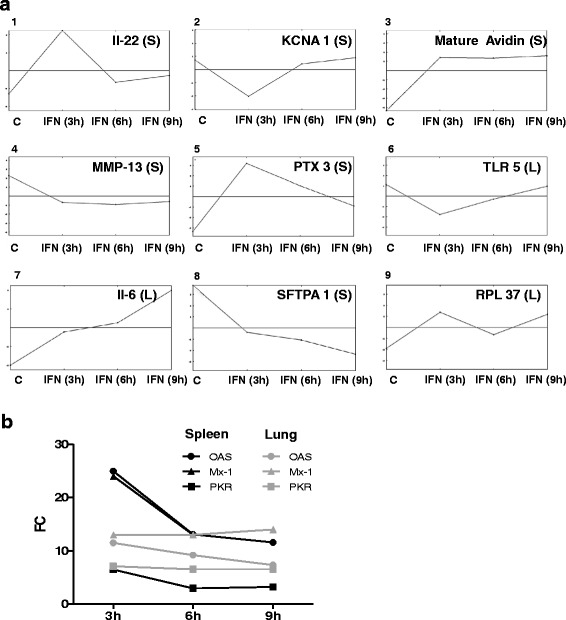
IRGs exhibit different expression profiles. **a** MEV analysis of genes with differential expression after 3 h identified nine different groups of expression profiles over the three sampling time points. Shown are characteristic examples for each group with gene name and tissue (S for spleen, L for lung) for which the profile was found. **b** Comparison of profiles for Mx, OAS and PKR in spleen (*black symbols*) and lung (*grey symbols*)

The chosen experimental design with three different time periods of IFN exposition allows for the generation of expression courses for single genes over time. Therefore, all genes differentially expressed in the group exposed for 3 h were subjected to analysis by Multi experiment Viewer (MeV). In total 1844 (99%, spleen) and 921 (97%, lung) of all regulated genes were assigned to an expression profile. When the identified profiles were compared and highly similar profiles were grouped, a total of nine groups of expression profiles were found (Fig. 4a and Table 4). One third of the genes belong to profile 1 (solely up-regulated after 3 h) and one third to profile 2 (solely down-regulated after 3 h), showing again, that more than 60% of the regulated genes are exclusively regulated after 3 h. Profiles 3 and 4, which represent continuous up- and down-regulation over all time points, contain another quarter of regulated genes. Far less genes were assigned to profiles 5 to 9, which contain genes with a diverse additional regulation at 6 and 9 h. Remarkably, the classical IRGs Mx (*Mx1*), OAS (*OAS*) and PKR (*EIF2AK2*) have identical expression profiles, they all show a continuously elevated abundance and were assigned to profile 5 in the spleen and profile 3 in the lung (Fig. 4b).

**Table 4:** Expression profiles identified by MeV. Shown are the numbers of genes assigned to the different clusters (see Fig. 4a), subdivided into cIRGs and nIRGs and the respective tissues

Expression profile	# of genes	cIRGs		nIRGs	
		Spleen	Lung	Spleen	Lung
1	900	139	59	462	240
2	798	52	23	543	180
3	317	35	63	108	111
4	399	28	5	319	47
5	156	29	4	57	66
6	38	3	2	31	2
7	39	0	7	8	24
8	20	1	0	19	0
9	98	2	28	8	60

Profile 8, representing a continuous down regulation, was not observed for IRGs in the lung. Interestingly, profile 7 which shows a stair-like up-regulation after 3 and 9 h, contains only few genes, but these have a stronger representation in the lung than in the spleen (31 genes vs. eight genes corresponding to 3.3 vs. 0.4% of all genes). The same applies to the undulating profile 9, which represents genes upregulated after 3 and again after 9 h as only 0.5% of splenic but almost 10% of the regulated lung genes belong to this profile. Overall, expression profiles for the majority of differentially expressed genes in spleen and lung were quite similarly distributed. Of note, for minor subgroups of genes tissue specific profiles were identified in the lung.

### IL6 protein level reflects RNA abundance

**Fig. 5:**
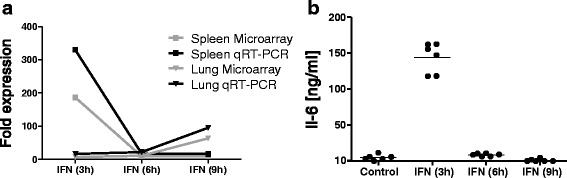
Validation of array results. **a** Comparison of detected fold changes for IL6 mRNA from the microarray experiment and qRT-PCR on identical samples. **b** Amount of IL6 protein in plasma samples of IFN treated and untreated animals

The cIRG *IL6* is well-known in mammals and in our array experiment it was both in the spleen and in the lung found among the genes with highest changes in abundance. To verify the microarray data the very same samples were additionally analysed by q-RT-PCR. As Fig. 5a shows, changes in RNA abundance detected by q-RT-PCR were slightly higher than those obtained from the microarray. But all lay in the same range and resulted in identical *IL6* expression profiles for spleen and lung.

RNA abundance is informative, but functional activity is only exhibited by proteins. To test whether RNA levels correspond to protein expression we used a bioassay to measure IL6 protein in plasma samples. As Fig. 5b clearly shows, significant amounts of IL6 were only detected in plasma samples taken 3 h after IFN treatment. This peak expression reflects exactly RNA expression levels in the spleen. Hence either cytokines in the spleen are rapidly released into the blood or the liver as main producer for IL6 reacts like the spleen. The lung’s *IL6* expression profile differs substantially and possible lung derived IL6 acts more locally.

### Gene ontology and pathway analyses

**Fig. 6:**
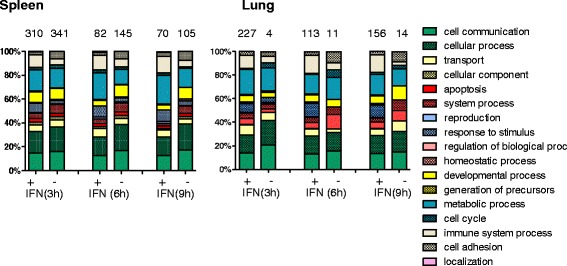
Gene ontology of differentially expressed genes. Differentially expressed genes with an FC of at least ± 3 were subjected to Panther gene ontology. Shown are identified biological processes and their percental distribution between genes with higher (+) and lower (-) mRNA abundance after IFN injection in spleen and lung

In order to analyse which biological processes were influenced by type I IFN treatment, Gene ontology (GO) analysis was performed looking at different aspects. Firstly, all genes with an at least 3fold change in abundance were subjected to Panther GO analysis (Fig. 6). Hereby, no significant differences between up- and downregulated genes and over time could be identified. In each data set “cell communication”, “cellular process” and “metabolic process” represented the functional classes with the highest percentage of affiliated genes. This was followed by “developmetal process” and “immunological process”. In addition, there were only minor differences between lung and spleen though in the lung proportionally more genes belong to “apoptosis”.

Next, GO analysis was performed with separated data sets for cIRGs and nIRGs, which were differentially expressed either in spleen and lung or only the spleen or only the lung. (Additional file 5: Figure S1A). Thereby neither examination of “molecular process” nor “biological process” nor “cellular component” revealed significant differences between cIRGs and nIRGs or between lung and spleen. In all data sets analysed for biological processes the subgroups “cell communication”, “cellular process” and “metabolic process” were most strongly represented. Among cIRGs the proportion of genes assigned to “Immune system process” was slightly higher than among the identified nIRGs. A more detailed analysis of the selected data sets for “cellular process” showed that in all groups “cell communication” is the most prominent subclass, mostly comprising genes which were assigned to “cell cell signalling” (Additional file 5: Figure S1B).

Differentially expressed genes were also subjected to Pathway Express. Table 5 shows the 30 most significantly regulated pathways in spleen and lung after 3 h. In the lung “Phosphatidyl-Inositol-Signalling system” was the pathway with the highest level of significance being assigned a corrected gamma p-value of 6,7×10^−11^. Phosphatidyl-Inositol-Signalling is of major importance for intracellular signalling and is involved in a multitude of cellular processes like cell cycle progression, growth, motility, adhesion and survival [47]. Among the regulated genes is *PTEN* (phosphatase and tensin homolog) with a 13.5-fold upregulation. *PTEN* acts as a phosphatidylinositol-3,4,5-trisphosphate 3-phosphatase and is known as a tumor suppressor gene. It negatively regulates PI3K induced Akt-Signalling and prevents uncontrolled, too fast cell divisions.

**Table 5:** KEGG Pathway Express analysis

	Spleen				Lung			
Pathway name	Rank	Impact factor	input genes/genes in pathway	corrected gamma *p*-value	Rank	Impact factor	input genes/genes in pathway	corrected gamma *p*-value
Leukocyte transendothelial migration	1	138.9	16/119	**6.56E-59**	52	3.1	7/119	0.190507
Cell adhesion molecules (CAMs)	2	123.4	15/134	**3.30E-52**	54	2.9	7/134	0.209069
Phosphatidylinositol signaling system	21	5.7	6/76	0.02196	1	27.5	7/76	**3.22E-11**
Jak-STAT signaling pathway	3	16.0	32/155	2.00E-06	2	18.7	24/155	**1.55E-07**
Toll-like receptor signaling pathway	4	14.9	24/102	5.22E-06	3	17.2	19/102	**6.12E-07**
Circadian rhythm	11	8.5	3/13	0.00192	4	17.1	1/13	**7.32E-07**
Graft-versus-host disease	5	14.4	6/42	**8.59E-06**	7	11.7	6/42	1.02E-04
Pathways in cancer	6	13.6	63/330	**1.89E-05**	5	13.4	39/330	2.19E-05
Complement and coagulation cascades	31	4.0	11/69	0.08904	6	12.7	13/69	**4.11E-05**
Axon guidance	7	12.4	30/129	**5.31E-05**	49	3.2	9/129	0.174755
Bladder cancer	8	11.7	14/42	**1.05E-04**	35	3.9	5/42	0.098477
Hematopoietic cell lineage	9	11.6	17/87	**1.19E-04**	9	8.5	11/87	0.001957
MAPK signaling pathway	10	10.9	44/272	**2.21E-04**	24	4.7	19/272	0.052229
TGF-beta signaling pathway	35	3.8	13/87	0.10746	8	8.9	13/87	**0.001400**
ECM-receptor interaction	12	7.9	20/84	0.00334	10	8.1	13/84	**0.002819**
Type II diabetes mellitus	22	5.6	7/45	0.02363	11	8.0	7/45	**0.003114**
Long-term depression	13	7.8	17/75	**0.00361**	44	3.2	2/75	0.170290
Calcium signaling pathway	38	3.7	23/182	0.11330	13	7.6	17/182	**0.004315**
Type I diabetes mellitus	14	7.5	8/44	**0.00464**	12	7.6	6/44	0.004176
Adherens junction	15	7.0	11/78	**0.00718**	33	4.0	3/78	0.091871
Allograft rejection	16	7.0	7/38	**0.00741**	15	6.7	5/38	0.009661
Epithelial cell signaling in Helicobacter pylori infection	17	6.9	15/68	**0.008295**	16	6.4	9/68	0.011897
Apoptosis	25	5.5	15/89	0.02658	14	6.8	11/89	**0.008871**
Amyotrophic lateral sclerosis (ALS)	18	6.4	10/56	**0.01234**	39	3.6	5/56	0.123832
Focal adhesion	19	6.2	35/203	**0.01415**	34	4.0	17/203	0.095007
Small cell lung cancer	20	6.0	18/86	**0.017727**	30	4.1	9/86	0.083372
Systemic lupus erythematosus	26	5.4	10/144	0.02984	17	5.7	7/144	**0.021797**
GnRH signaling pathway	23	5.6	18/103	**0.02426**	32	4.1	8/103	0.085545
PPAR signaling pathway	24	5.6	14/70	**0.02482**	-	-	-	-
Long-term potentiation	41	3.3	11/73	0.15896	19	5.4	5/73	**0.028017**

Shown are the 30 most significantly affected pathways in spleen and lung ranked according to their corrected gamma *p*-value (the value which was used for the ranking is shown in bold letters); “Rank” is the rank of a pathway among all significantly affected pathways according to its impact factor; the “Impact factor” of a pathway is calculated from a probabilistic term considering the proportion of differentially regulated genes on the pathway and gene perturbation factors of all genes in the pathway [46]

In the spleen “leukocyte transendothelial migration” and “cell adhesion molecules” are ranked number one and two, reflecting the migratory activation of leukocytes in this central organ of the adaptive immune system. They are followed by “JAK-STAT-“and “TLR-signalling”, two pathways which play an essential role for the activation of the innate immune system and are also among the top four pathways in the lung. Three hours after IFN application *TLR3* and *TLR15* were upregulated in both lung and spleen. *TLR3*, the intraendosomal receptor for double stranded RNA, showed the strongest increase in abundance of all TLRs with an FC of +10 in the lung and +6 in the spleen and stayed upregulated at 6 and 9 h (lung: FC + 6 and +7; spleen FC +4 and +4). *TLR15* is unique to reptiles and birds and binds a yeast component [48]. In contrast to the short and relatively weak upregulation in the spleen, we observed a continuous upregulation in the lung (FC +4, +2 and +5 after 3, 6 and 9 h). Interestingly, *TLR5*, the receptor for flagellin, was clearly downregulated in both tissues (FC –18 in the lung), while no regulation was observed for *TLR21*, the CpG motive binding chicken homologue of *TLR9*. In addition to the TLR genes themselves, the important signalling molecules *TRAF3* and *IRF7* were upregulated, which after TLR stimulation lead to the observed transcription of inflammatory cytokines (*IL1ß*, *IL6, IL12*) and chemokines (*IL8,* MIP family members and *RANTES/CCLi4*).

**Fig. 7:**
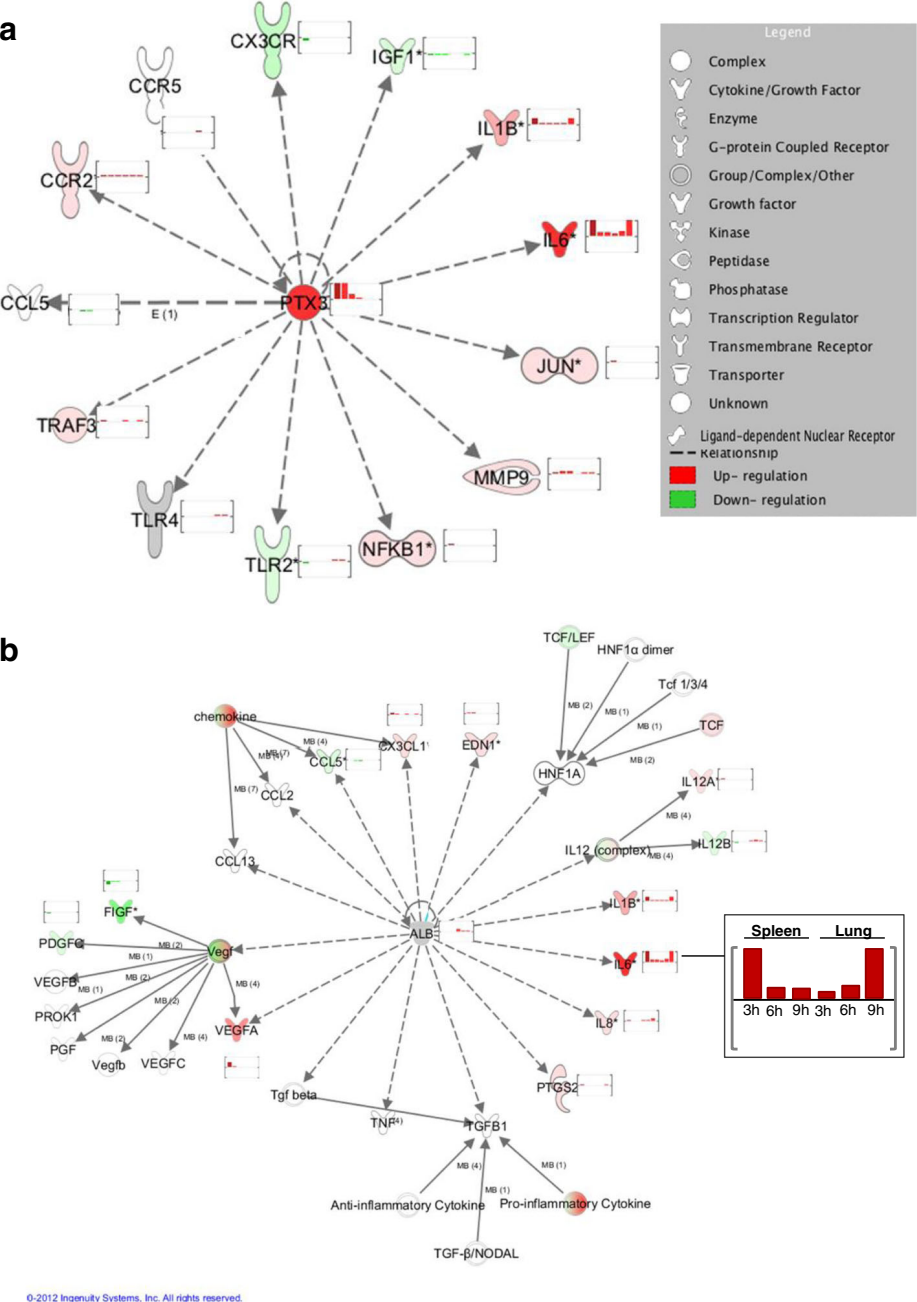
Multilayer interaction of differentially expressed genes. IPA network analysis demonstrating gene interactions with the upstream regulators PTX3 (**a**) and Albumin (**b**). Genes with higher mRNA abundance in the IFN treated animals are shown in *red*, genes with lower mRNA abundance in the treated animals in *green*. The small diagrams next to each differentially expressed gene display expression (FC) at the different time points. The magnification shows the expression course for IL6

Another possibility to gain information about the connection of differentially expressed genes is a network analysis. Therefore, array data were subjected to the Interactive Pathway analysis of complex ‘omics data (IPA) from Ingenuity®. Several genes with interesting features were manually chosen as so called “upstream regulators” for a causal network analysis in order to identify genes which are directly or indirectly connected to the regulator. Exemplary, the networks for the highly upregulated cIRGs *PTX3* and *IL22*, the strongly downregulated cIRG *SFTPA1* and the nIRG *ALB* (albumin) are shown (Fig. 7 and Additional file 6: Figure S2).

With a 685fold upregulation (spleen, 3 h), *PTX3* is the gene with the highest FC of all tissues and timepoints. As Fig. 7a shows, all of the known mammalian interactors of PTX3 were likewise regulated after IFN treatment in chickens, including several cytokines (*IL1* and *IL6*), TLRs (*TLR2* and *TLR4*) and transcription factors (*NFκB1* and *JUN*). Besides, the identified interactors represented both cIRGs and nIRGs.

A nIRG for which an IPA network was found is Albumin (*ALB*) (Fig. 7 b). Albumin showed a 10fold upregulation in the lung after 3 h but was not affected in the spleen. IPA identified 14 significantly regulated genes to interact with albumin, among them *IL1, IL6, IL12* and *VEGF*. And as for *PTX3* interacting genes represent both cIRGs and nIRGs.

*IL22* was 34-fold upregulated in the spleen (3 h) and 3-fold upregulated in the lung (9 h). From the 28 (out of 34) annotated interactors of *IL22*, 22 were significantly regulated by treatment with IFNα, thereby containing 14 cIRGs and 8 nIRGs (Additional file 6: Figure S2A). Among the connected genes were both, inflammatory cytokines (*IL6, IL1, IL18*) and regulators of an immune response (*SOCS3, CD274/PD-L1*).

Surfactant protein A1 *(SFTPA1)* is highly interesting, as it shows the strongest decrease in abundance with a 436fold downregulation in the spleen and exhibits its function through reduced expression. IPA identified 49 interacting genes, from which 31 were annotated on the microarray. Additional file 6: Figure S2B shows that about half of the known mammalian interactors were significantly affected in the chicken, nine of them being cIRGs and 13 belonging to the identified nIRGs.

Overall, IPA network analysis, a tool based on human, mouse and rat data, demonstrates that networks which were initially described in mammals are to a large part also activated in chickens and connected genes are quite equally distributed between cIRGs and nIRGs.

### Cytokines and chemokines

A main effect of type I interferon on cells is to induce an antiviral state e.g. by the induction of Mx, PKR and OAS. Yet, GO analysis and Pathway express show that cell communication and cytokine signalling are as well strongly activated. As we added a multitude of probe sets for cytokine and chemokine genes and their receptors to the applied array-system, a closer examination of these cellular communicators was possible. About 20% of the Top30 genes in the spleen belong to cytokines and chemokines and *IL6* and *CCLi3/K203*, a chicken CC-chemokine and putative homologue to human CCL16, were among the genes with strongest upregulation in spleen (*IL6* + 186; *K203* + 170) and lung (*IL6* + 67; *K203* + 16).

**Fig. 8:**
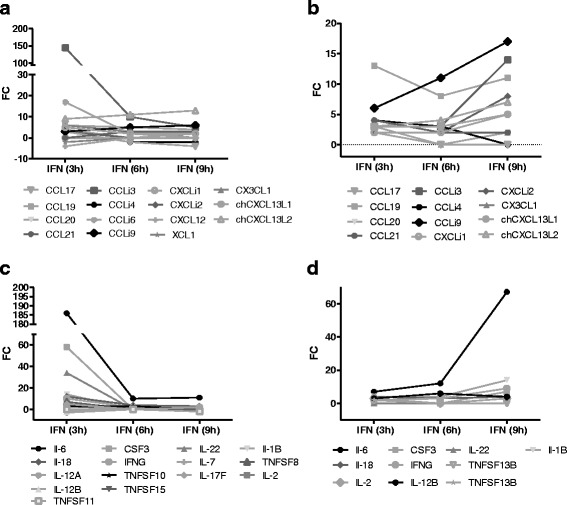
Tissue and time specific expression profiles for chemokines and cytokines. Fold expression of differentially expressed chemokines (**a** + **b**) and cytokines (**c** + **d**) after IFN injection in the spleen (**a** + **c**) and the lung (**b** + **d**) in relation to control birds

As Table 6 shows, IFN treatment leads to expression changes of both inflammatory and homeostatic chemokines. Among the regulated inflammatory chemokines were members of the MIP family (*CCLi3, CCLi4*), the MCP family (*CCLi9*) and both IL8 orthologs (*CXCLi1* and *CXCLi2*). Among the homeostatic chemokines, expression of all four CCL family representatives (*CCL17, 19, 20, 21*), *CXCL12* and the *L1* and *L2* isoforms of *CXCL13* were regulated. Interestingly, the majority of chemokines in the spleen shows an early increase in abundance after 3 h (ten out of 15) with close to control levels after 9 h, while the opposite is seen in the lung, where expression levels for the majority of chemokines rise up to 9 h (Fig. 8a and b). For *CCLi3 (K203)*, *CXCLi1 (K60)* and *CXCLi2 (IL8)* this results in inverted profiles in spleen and lung, demonstrating again a highly tissue specific response.

**Table 6:** Fold expression of differentially expressed chemokines and chemokine receptors after IFN treatment

	Spleen			Lung		
Gene symbol	IFN (3 h)	IFN (6 h)	IFN (9 h)	IFN (3 h)	IFN (6 h)	IFN (9 h)
CCLi3	145	10	5	3	3	14
CCLi6	17	2	-	-	-	-
CCLi9	3	5	6	6	11	17
CXCL13L2	9	11	13	3	4	7
CCL19	6	5	4	13	8	11
CXCLi2	3	-	-	2	2	8
CX3CL1	6	3	-	2	-	2
CXCLi1	5	2	2	2	2	5
CXCL13L1	-	-	2	3	3	5
CCL20	4	-	-	2	-	2
CCL21	-	3	3	4	2	2
CCLi4	3	−2	−2	4	3	-
CCL17	3	−2	−4	3	-	-
XCL1	−2	-	-	-	-	-
CXCL12	−4	-	-	-	-	-
CXCR4	5	-	-	-	-	-
CCRa	2	3	2	4	3	3
CCR7	-	-	-	2	-	2
CCR8	2	-	-	-	-	-
XCR1	−2	-	−2	-	-	-
CXCR7	-	-	-	−2	-	−2
CCR6	−3	-	−2	-	-	-
CX3CR1	−5	-	-	-	-	-

Even more clear-cut is the picture for the regulated cytokines (Table 7 and Fig. 8c and d). Altogether IFN treatment altered the abundance of 15 cytokines and almost all highly induced cytokine genes (*IL6, CSF3, IL22, IL1* and *IFNγ*) show the above described inverted expression profiles in spleen and lung with highest abundances after 3 h in the spleen and 9 h in the lung.

**Table 7:** Fold expression of differentially expressed cytokines and cytokine receptors after IFN treatment

	Spleen			Lung		
Gene Symbol	IFN (3 h)	IFN (6 h)	IFN (9 h)	IFN (3 h)	IFN (6 h)	IFN (9 h)
IL6	186	10	11	7	12	67
CSF3	58	-	-	-	-	7
IL22	34	-	3	-	-	3
IL1B	14	-	-	4	4	14
IL18	12	4	3	4	3	4
IFNG	10	2	2	2	3	9
CD40	8	3	-	-	-	-
TNFSF8	7	-	-	-	-	-
IL12B	−2	-	-	3	6	4
IL2	5	-	-	3	-	3
IL17F	5	-	2	-	-	-
TNFSF15	5	-	-	-	-	-
FAS	3	-	-	3	-	2
IL12A	3	-	-	-	-	-
TNFSF10	3	3	-	-	-	-
TNFSF13B	-	-	-	2	-	-
TNFSF11	-	-	−2	-	-	-
IL7	−3	-	-	-	-	-
TRAIL	−3	-	-	-	-	-
IL1RL1	15	5	3	14	3	3
IL13RA2	13	7	6	14	12	18
TNFRSF8	9	3	4	-	-	-
IL1R2	7	3	-	3	-	-
TNFRSF11B	3	-	-	5	-	-
CSF3R	-	4	4	-	2	2
IL20RA	4	3	-	3	-	-
CSF2RB	4	-	-	3	-	2
IL2RA	4	-	-	-	-	2
IL23R	4	2	-	-	-	-
CSF2RA	3	2	-	3	3	3
IL10RB	3	-	-	2	-	-
TNFRSF1B	3	-	-	2	-	-
IL21R	2	-	-	3	-	-
TNFRSF1A	-	-	-	2	-	3
IFNGR1	3	-	-	-	-	-
IL4R	3	-	-	-	-	-
IL1R1	3	-	-	-	-	-
IFNAR1	2	-	-	2	-	-
TNFRSF13C	2	-	-	-	-	-
IL7R	2	-	-	-	-	-
IL10RA	2	-	-	-	-	-
IL12RB2	−2	-	-	-	-	-
IL28RA	−2	-	-	-	-	-
IL1RL2	-	−2	−2	-	-	-
IL11RA	−3	-	-	-	-	-

## Discussion

### Methodical aspects

Though type I IFNs were first described in the chicken, the vast majority of available information about these cytokines is derived from human and mouse studies. Recently it became clear that the pleiotropic functions of type I IFNs are mediated by the regulation of a huge number of genes. As the chicken’s immune system differs significantly to that of human and mouse we aimed for a better understanding of the chicken’s response to IFNα. The performed database analysis identified more than 1400 human/murine IRGs which are also present in the chicken genome. As the existence of such a gene does not necessarily argue for its regulation by IFN, birds were intravenously treated with IFNα and changes in gene expression were analysed in lung (the target tissue of many important pathogens like AIV, IBV, NDV, APEC) and spleen (the most prominent secondary lymphatic tissue in birds). The setting evidently differs from a naturally occurring lung infection, where a local immune response with production of type I IFN would be induced first, which then could become systemic. However, it avoids the mixture of effects seen in a natural immune response, where several cytokines and effector molecules are induced in parallel.

In order to apply biologically relevant amounts of IFN, we first determined the pharmacokinetic profile of recombinant chIFN in chickens. With a terminal half-life of approximately 36 min it is clearly shorter than the half-life reported for human IFNα (4-6 h) or human IFNβ in humans (1-2 h) [49], an observation which can possibly be attributed to the higher metabolic activity in birds. Using an application setting based on these data we were able to obtain a constant IFN level during the experiment comparable to levels observed during natural occurring viral infections [20].

Heatmap analysis of the performed arrays already showed two things: firstly, strong effects of IFN on gene expression in both tissues studied with a clear separation of treated and control birds and secondly, explicit differences between spleen and lung. As expected a closer look at the differentially expressed genes showed a strong induction of IRGs *Mx, OAS* and *PKR*, thus proving the reliability of the experiment. Regarding the time point of expression the vast majority of differentially expressed genes (86% in the spleen and 80% in the lung) was found 3 h after IFNα application.

Surprisingly, we observed relatively little concordance between our *in silico* and in vivo analyses, with many IRGs identified in the database search being not differentially expressed in the array and many differentially expressed genes having not been found in the database. In agreement with our observation Giotis and colleagues analyzed IRGs in IFNα treated chicken embryo fibroblasts (CEF) and they also identified many potential chicken IRGs with no equivalent in the Interferome database [29]. Partly, annotation problems could be a reason as entries in the database and the chicken genome use a different nomenclature, hence mammalian IRGs were not found on the array. Nevertheless, it is very unlikely that this applies but for all 70% of the genes without mammalian equivalent. The finding of so many additional differentially expressed genes might have been facilitated by the usage of a strong stimulation and the high sensitivity of the array. Furthermore, we analyzed both increased and reduced expression of target genes, while many other approaches were only focused on positively affected, so called “Interferon Stimulated Genes” (ISGs) [13, 29]. Among the 30 most affected genes in the spleen were eight with reduced expression and only one of them (*SFTPA1*) was also identified in the database search.

Other reasons for the discrepancy between *in silico* and array results might be time-, tissue- and species specificity. Database entries comprise a multitude of tissues and cells with different times of IFN exposition [12]. Our analysis demonstrates large differences between the two examined tissues and three examined time points. Hence, testing of additional tissues and time points might reveal further differentially expressed genes and a larger overlap with the database search. And despite the general conservation of the IFN pathway some IRGs might be species specific.

### cIRGs and nIRGs

The fact that so many differentially expressed genes had not been found in the database search suggested to discriminate between common (database) IRGs (cIRGs) and newly (only in the microarray) identified IRGs (nIRGs). To increase the probability that identified nIRGs are truly regulated by interferon, only genes differentially expressed after 3 h of IFN exposition were considered as IRGs and used for downstream analyses as at later time points secondary effects could not be excluded. For instance *IL6* expression was strongly induced and high amounts of protein were found after 3 h (Fig. 5). As *IL6* itself induces an inflammatory reaction, at later time points genes could be regulated by *IL6* instead of IFN.

Another approach to compare cIRGs and nIRGs was the search for ISRE and GAS elements. Unfortunately, an automated search did identify only a very low number of both of them in the promotor region of the analysed genes and did not reveal clear differences between both groups of IRGs and control genes. The low overall coverage is possibly caused by weak sequence conservation between chicken on the one hand and mouse and man on the other. Additionally, due to technical limitations we were only able to screen a portion of functional ISRE and GAS elements and may have missed chicken specific ISRE and GAS elements or so far unidentified ISRE and GAS elements which are conserved across species.

More successful was the individual screening of the 30 most strongly affected genes, were with one exception in every gene an ISRE or GAS element was identified. Surprisingly, in contrast to mammalian data [41] far more GAS than ISRE elements were found, but it has already been shown that type I IFN induced transcription factors are able to activate GAS elements [44]. So, despite its limitations, the promotor analysis revealed identical motive distribution in cIRGs and nIRGs.

IPA interaction shows that upstream regulators interact equally with cIRGs and nIRGs and the GO and pathway distribution of both groups is largely identical. The fact that “Immune system process” was slightly stronger represented among cIRGs than in nIRGs, might be caused by a stronger focus on immune genes of the used databases. Overall, we think it is very likely that expression of the identified nIRGs is regulated by IFN.

### Tissue and time specificity

The heatmap analysis had already pointed to a tissue specific reaction to IFN exposure. The clear separation of the 3 h expression pattern in the spleen points to a fast peaking response while the larger similarities between 3, 6 and 9 h in the lung suggest a slower and more sustained reaction of this organ. Changes in RNA abundance support this observation, as these were in general stronger in the spleen, where also twice as many differentially expressed genes were identified. For instance absolute changes of *Mx, OAS* and *PKR* abundance were clearly higher in the spleen and the expression profiles of these IRGs show a strong 3 h peak in the spleen versus a continuous up regulation (plateau) in the lung.

Though the receptor for type I IFN is ubiquitously expressed [50] and theoretically every cell can react to IFN stimulation, the more homogenous tissue composition of the spleen might lead to more intense variations than in the heterogeneous lung, where the cartilaginous parts might react differently from epithelium and lung associated immune cells. In addition, immune cells may show a more pronounced reactivity per se and this difference might have been increased as we, on purpose, did exclude large lymphoid structures in lung samples by avoiding major BALT regions during sampling.

Tissue specificity is also underlined by pathway analysis, as the most strongly affected pathways differ in spleen and lung. Interestingly, the two main pathways in the spleen “Leukocyte transendothelial migration” and “Cell adhesion molecules” were also the most affected after infection with IBDV [51]. As one would expect, IBDV infection led to the expression of type I IFN, but in parallel also IFNγ as well as many other cytokines were induced. Even though the stimuli were heterogeneous in the IBDV experiment the reaction pattern of the spleen was similar to a sole stimulation with IFNα, supporting the idea of a tissue specific reaction.

Tissue specificity is not only presented by the diversity of affected genes but also by time specific expression patterns. Generation of expression courses for all differentially expressed genes revealed that two thirds of the genes affected in both tissues are solely up- or downregulated after 3 h of IFN exposition. However, profiles with up regulation after 9 h are proportionately much stronger represented in the lung than the spleen. This is for instance shown by expression profiles of regulated chemokine and cytokine genes. In many of these profiles the spleen reacts early and the lung reacts late, leading to opposite profiles e.g. for inflammatory chemokines (*CCLi3* (*K203*), *CXCLi1* (*K60*), *CXCLi2* (*IL8*)) and inflammatory cytokines (*IL1, IL6*). Interestingly, this time specific reaction of IRGs was also observed in a transcriptome analysis of cultured CEF [29] and hence seems to be more of a general phenomenon than restricted to lung or spleen.

### Conserved interaction of selected upstream regulators

Network analysis with selected “upstream regulators” suggests a widely conserved interaction pattern for chicken IRGs as many of the known mammalian interactors are also affected in the chicken.

*PTX3*, the gene with the highest fold change (FC), is a member of the pentraxin superfamily like the acute phase proteins CRP (C reactive protein) and Serum Amyloid P Component (SAP) and acts as Pattern Recognition receptor (PRR). During inflammatory processes PTX3 is rapidly produced by macrophages, dendritic cells, fibroblasts and epithelial cells [52]. It is known to activate the classical pathway of the complement system and facilitates pathogen recognition by DCs and MQs [52–54]. By binding to hemagglutinin it is also able to inhibit infectivity of influenza viruses [55]. In mammals PTX3 is known to directly and indirectly influence cytokines and their receptors, growth factors, G-protein coupled receptors, peptidases and transcription factors [56]. As we found regulation of *IL1, IL6, IGF1* and also *NFkB* and *Jun*, chicken PTX3 seems to have a similar mode of action.

Serum albumin is a “negative acute-phase protein”, which means its abundance decreases during an acute-phase-response, probably to save amino acids for positive acute phase proteins [57, 58]. The IFNα induced upregulation of albumin in the lung strongly argues for an active function of this protein as indicated by the connection with several cytokines and chemokines. It is known that human albumin protects tissue against oxidative stress by blocking reactive oxygen species (ROS) from neutrophils [59, 60]. Hence, albumin could protect the lung against deleterious side effects of IFN.

For Surfactant protein A1 *(SFTPA1)* we found the strongest decrease of RNA abundance. Reduced expression of *SFTPA1* was also observed in infection studies with chickens using ILTV (Infectious Lanryngotracheitis Virus) and Avian Influenza Virus H9N2 [61, 62], possibly mediated by virus induced IFN. It was shown that suppression of the P38 MAPK and the PI-3 kinase pathway can downregulate *SFTPA1* [63, 64] and especially for the PI-3 kinase pathway we observed a very strong activation in spleens and lung. Two thirds of the IPA annotated interactors were also found in the chicken and among them are several proinflammatory cytokines (IL1, IL6), whose production can be suppressed by SFTPA1 [65]. Hence reduced SFTPA1 abundance allows for a higher induction of proinflammatory cytokines and a stronger IFN induced immune response.

IL22, a strongly upregulated cIRG is a member of the IL10 superfamily. Being pro-regenerative, pro-proliferative and anti-apoptotic its main function is to modulate the tissue response during an inflammatory reaction [66]. The interactors for IL22 which we identified in our data set follow the idea of a modulating cytokine as both inflammatory factors and regulators of an immune response were found. While IL22 is exclusively produced by leukocytes of the innate and adaptive immune system, the IL22R is mostly expressed on epithelial cells [67]. In mice a close interaction of IL22 and IFNα and IFNλ, respectively was demonstrated for lung and gut and this interplay between IL22 and interferon seems also be true for the chicken.

### Sensitization and desensitization

For several species it was shown that pathogen recognition induces the production of type I IFN and induces a multitude of effector mechanisms. In addition, exposure of cells to IFN does sensitize the cells for further detection of pathogens [6]. In our studies this was also demonstrated for chicken cells as IFN treatment upregulated TLRs for RNA and yeast detection, the RNA detecting OAS and the cytosolic NOD-like receptor *NLRC5*. NLRC5 shows a close evolutionary relationship to NLRC1 and NLRC2 and hence is probably involved in detection of bacterial infections [68].

In mice it was shown that in most cells pathogen sensing leads first to the expression of IFNβ, which then induces an amplification step IFNα [7]. We did not find upregulation of mRNA for type I IFNs, which might be caused by the artificial setting or a different induction mechanism in the chicken. In contrast to type I IFNs, IFNγ was upregulated in spleen and lung and showed the described inverted expression profiles. This shows that type I IFN is not only a strong effector of the innate immune response but by inducing IRGs like *IFNγ*, *IL18, IL2* and *CXCL13* does also activate adaptive immunity.

IFN signalling can induce a powerful inflammatory immune response and therefore needs to be tightly controlled. Once an IFN response is initiated desensitization of cells is induced in parallel by several mechanisms. One proposed mechanism is downregulation of the IFN receptor, but we found a slight upregulation of *IFNAR1* RNA after 3 h. In mammals, the action of SOCS (suppressor of cytokine signalling) proteins, which inhibit JAK-STAT signalling, is well described as early desensitizing mechanism. The same seems to be applicable to chickens as a significant upregulation of *SOCS1* and *SOCS3* in spleen and lung, with a strong 3 h peak in the spleen and a plateau in the spleen was obseved. USP18 does maintain long-term desensitization by either removing ISG15 conjugated proteins or intracellular binding to IFNAR2 [69, 70]. Strong upregulation of *USP18* in spleen and lung after 3 h demonstrates the conservation of negative control mechanisms of the type I IFN response in chickens.

## Conclusion

The application of a constant, physiological amount of IFNα over 9 h affected a multitude of IRGs in spleen and lung. We identified many different expression profiles and found that induction/suppression of IRGs is both tissue and time specific. It became apparent that type I IFN does not only induce antiviral mechanisms but also mediates a multitude of different functions. As we found little overlap of our data with existing databases, we here provide a large new dataset for further research, both to expand the data set by further tissues and time points and to examine single IRGs for their individual function. Our study provides the basis for the potential identification of new antiviral mechanisms with broader screening approaches using RNA interference or gene editing technologies like CRISPR/Cas9.

## Additional files

Additional file 1: Table S1. Sequences of ISRE/GAS promotor elements used for large scale screening in chicken genes. (DOCX 15 kb)

Additional file 2: Table S2. Common ISGs Extensive comparative database analysis to relate known mammalian ISGs to annotated chicken genes using entries in INTERFEROME, the ISG-database, KEGG, Reactome and several publications all annotated 13,353 genes on a customized laboratory internal Agilent 4x44K chicken Genome microarray. (XLSX 339 kb)

Additional file 3: Table S3. Overview on pharmacokinetic parameters of rec chIFNα in LSL chickens. (DOCX 16 kb)

Additional file 4: Table S4. Number of ISRE and/or GAS motifs in chicken genes with highest mRNA abundance after IFN injection in spleen and lung tissue. (DOCX 22 kb)

Additional file 5: Figure S1. Gene ontology after IFN injection. Assignment of cIRGs and nIRGs which were differentially expressed in both spleen and lung or only one of the tissues to GO terms. Numbers in the pie charts indicate the quantity of genes in each subgroup. Shown are the GO terms “Response to stimulus” and “Immune system process” and “Immune response” as a part of these two (A) and “Cellular process” and its subterm “Cell communication” (B). (PPTX 15674 kb)

Additional file 6: Figure S2. IPA network analysis for IL22 and SFTPA1. Gene interactions of IL22 (A) and SFTPA1 (B) obtained by IPA. Genes with higher mRNA abundance in the IFN treated animals are shown in red, genes with lower mRNA abundance in the treated animals in green. The small diagrams next to each differentially expressed gene display expression (FC) at the different time points. (PPTX 2050 kb)

## Abbreviations

APEC: Avian pathogenic *E.coli*
BALT: Bronchus associated lymphoid tissue
cIRG: common IRG
CRP: C reactive protein
DC: Dendritic cell
FC: Fold change
FDR: False discovery rate
GAS: γ-activated sequences
GO: Gene ontology
HPAI: highly pathogenic avian influenza
IFIT: IFN induced transmembrane protein
IFN: Interferon
IL: Interleukin
IRG: Interferon regulated gene
IRNAR: Interferon-α/β receptor
ISRE: interferon-stimulated response element
MeV: Multi experiment Viewer
MQ: Macrophage
Mx1: Myxovirus resistance 1
NDV: Newcastle disease virus
nIRG: newly identified IRG
OAS: 2’-‘5-oligoadenylate synthetase
PKR: IFN-inducible double-stranded RNA-dependent protein kinase
PRR: Pattern Recognition receptor
SAP: Serum Amyloid P Component
TLR: Toll like receptor
