# Supplementary material for: Tissue and time specific expression pattern of interferon regulated genes in the chicken
Source: BMC Genomics. 2017 Mar 28;18:264. doi: 10.1186/s12864-017-3641-6 (PMC5371264; doi:10.1186/s12864-017-3641-6)
Supplement: Supplementary file 1 — Sequences of ISRE/GAS promotor elements used for large scale screening in chicken genes. (DOCX 15 kb) [file 12864_2017_3641_MOESM1_ESM.docx]

| ISRE |
| --- |
| TAGAAAATGAAACCA |
| GGGAAACCGAAACTG |
| AGTTTCACTTTCCT |
| AGTTTCACTTTCCC |
| TAGAAACTGAAACAG |
| TGGAAAGTGAAACCT |
| GGGAAAATGAAACTC |
| GGGAAAATGAAACTG |
| GGGAAAACGAAACTG |
| AGGAAATAGAAACTT |
| AGGAAAAGGAAACTG |
| GAGAAACCGAAACTA |
| TGGTTTCAGTTTTCC |
| TGGTTTCATTTTCTA |
| CAGTTTCTGTTTCCT |
| CAGTTTCGGTTTCCC |
| GAGTTTCATTTCTTC |
| AGGTTTCGTTTCTGC |
| CTGTTTCAGTTTCTA |
| AGGTTTCACTTTCCA |
| GAGTTTCATTTTCCC |
| CAGTTTCATTTTCCC |
| CGCTTTCGTTTCCTC |
| CAGTTTCGTTTTCCC |
| AAGTTTCTATTTCCT |
| CAGTTTCCTTTTCCT |
| TATGTTTCGGTTTCTC |
| TGGTTTCGTTTCCTC |
| GAS |
| TTTCCCAGAAA |
| TTTCCGGGAAA |
| TTTCCTTGAAA |
| TTTCTGAGAAT |
| TTTCCTGTAAA |
| TTTCCCCGAAA |
| TTTCTCGGAAA |
| TTTCCCGTAAA |
| ATTCTCAGAAA |

**Supplementary Table 1** Sequences of ISRE/GAS Promotor elements used for large scale screening in chicken genes.
