# Supplementary material for: Tissue and time specific expression pattern of interferon regulated genes in the chicken
Source: BMC Genomics. 2017 Mar 28;18:264. doi: 10.1186/s12864-017-3641-6 (PMC5371264; doi:10.1186/s12864-017-3641-6)
Supplement: Supplementary file 3 — Overview on pharmacokinetic parameters of rec chIFNα in LSL chickens. (DOCX 16 kb) [file 12864_2017_3641_MOESM3_ESM.docx]

**Supplementary Table 3: Overview on pharmacokinetic parameters of rec chIFNα in LSL chickens**

Six weeks old LSL chickens received a single i.v. injection of 1x10^7^ Units for the biggest bird (animal 3) and a direct proportional weight adjusted dose for all others. Plasma samples were collected between 5 and 480 min after dosing and chIFNα levels were determined using a reporter-assay for chicken type I IFN. Pharmacokinetic parameters were calculated by Non Compartmental Analysis using Microsoft Excel®. Exposure assessment for determining Cl was performed using the linear-trapezoidal rule.

|  | **Weight [kg]** | **IFN-α [U]** | **Dose [U/kg]** | **C_max_ [U/ml]** | **T_max_  [min]** | **Cl  [ml/(hxkg)]** | **Vz  [l/kg]** | **T_1/2_**  **[min]** |
| --- | --- | --- | --- | --- | --- | --- | --- | --- |
| **Animal 1** | 0.615 | 0,83x10^7^ | 1,34x10^7^ | 18447 | 5 | 3288.60 | 3.08 | 38.99 |
| **Animal 2** | 0.689 | 0,93x10^7^ | 1,35x10^7^ | 14510 | 5 | 4028.20 | 3.30 | 34.06 |
| **Animal 3** | 0.744 | 1x10^7^ | 1,34x10^7^ | 16915 | 5 | 3747.82 | 3.21 | 35.57 |
| **Mean** | - | - | - | 16624 | 5 | 3688.21 | 3.20 | 36.21 |
| **SD** | - | - | - | 1985 | 0 | 373.39 | 0.11 | 2.53 |
| **CV [%]** | - | - | - | 11.94 | 0 | 10.12 | 3.38 | 7.00 |

C_max_: maximum concentration; T_max_: time point of maximum concentration; Cl: clearance; Vz: volume of distribution; T_1/2_: terminal elimination half-life
