# Supplementary material for: Tissue and time specific expression pattern of interferon regulated genes in the chicken
Source: BMC Genomics. 2017 Mar 28;18:264. doi: 10.1186/s12864-017-3641-6 (PMC5371264; doi:10.1186/s12864-017-3641-6)
Supplement: Supplementary file 4 — Number of ISRE and/or GAS motifs in chicken genes with highest mRNA abundance after IFN injection in spleen and lung tissue. (DOCX 22 kb) [file 12864_2017_3641_MOESM4_ESM.docx]

**Supplementary Table 3** Number of ISRE and/or GAS motifs in chicken genes with highest mRNA abundance after IFN injection in spleen and lung tissue. The scanned region ranged from 6kb upstream to 1kb downstream of the first exon.

Spleen Lung

| Gene symbol | # of Promotor-elements (ISRE/GAS) | 500 bp upstream  (ISRE/GAS) |
| --- | --- | --- |
| PTX3 | 0/7 | 0/1 |
| SFTPA1 | 0/12 | 0/1 |
| Avidin | 1/7 | 0/1 |
| Il-6 | 0/8 | 0/0 |
| LAO | 0/7 | 0/1 |
| LL | 0/12 | 0/1 |
| PSCA | 0/12 | 0/2 |
| K203 | 0/6 | 0/1 |
| KCNA1 | 0/5 | 0/1 |
| LYG2 | 0/6 | 0/0 |
| CSF3 | 0/3 | 0/1 |
| NEFH | 0/5 | 0/0 |
| CMBL | 0/12 | 0/1 |
| GCH1 | 0/9 | 0/1 |
| FSHR | 0/5 | 0/0 |
| ZPD | 0/13 | 0/2 |
| IRG1 | 0/9 | 0/0 |
| BATF3 | 0/11 | 0/0 |
| Il-22 | 0/4 | 0/0 |
| MAOA | 0/11 | 0/0 |
| HPS5 | 0/10 | 0/0 |
| ETV1 | 0/13 | 0/0 |
| MMP1 | 1/7 | 0/0 |
| LIPI | 1/9 | 0/0 |
| RPL37 | 0/9 | 0/0 |
| GBP | 0/5 | 0/0 |
| OASL | 0/0 | 0/0 |
| SPINK5 | 0/7 | 0/0 |
| TULP1 | 0/11 | 0/0 |
| Mx-1 | 1/16 | 0/1 |

| Gene symbol | # of Promotor-elements  (ISRE/GAS) | 500 bp upstream  (ISRE/GAS) |
| --- | --- | --- |
| TULP 1 | 0/11 | 0/0 |
| Il-6 | 0/8 | 0/0 |
| CMBL | 0/12 | 0/1 |
| Avidin | 1/7 | 0/1 |
| LAO | 0/7 | 0/1 |
| MSC | 0/5 | 0/0 |
| FKBP5 | 0/6 | 0/1 |
| TGM4 | 0/10 | 0/1 |
| LYG2 | 0/6 | 0/0 |
| BATF3 | 0/11 | 0/0 |
| GBP | 0/5 | 0/0 |
| RPL37 | 0/9 | 0/0 |
| IFIT5 | 1/9 | 1/0 |
| IRG1 | 0/9 | 0/0 |
| TLR5 | 0/11 | 0/0 |
| SEMA6D | 0/11 | 0/1 |
| GAL9 | 0/8 | 0/0 |
| Rd | 0/15 | 0/0 |
| MAOA | 0/11 | 0/0 |
| K203 | 0/6 | 0/1 |
| SAMD9L | 0/13 | 0/2 |
| Il-1RL1 | 0/15 | 0/0 |
| Mx-1 | 1/16 | 0/1 |
| SOCS3 | 1/9 | 0/2 , |
| OASL | 0/0 | 0/0 |
| RASD1 | 0/3 | 0/0 |
| ALB | 0/12 | 0/1 |
| SNAP25 | 0/19 | 0/3 |
| SPINK5 | 0/7 | 0/0 |
| GCH1 | 0/9 | 0/1 |
