# Supplementary material for: Tissue and time specific expression pattern of interferon regulated genes in the chicken
Source: BMC Genomics. 2017 Mar 28;18:264. doi: 10.1186/s12864-017-3641-6 (PMC5371264; doi:10.1186/s12864-017-3641-6)
Supplement: Supplementary file 5 — Gene ontology after IFN injection. Assignment of cIRGs and nIRGs which were differentially expressed in both spleen and lung or only one of the tissues to GO terms. Numbers in the pie charts indicate the quantity of genes in each subgroup. Shown are the GO terms “Response to stimulus” and “Immune system process” and “Immune response” as a part of these two (A) and “Cellular process” and its subterm “Cell communication” (B). (PPTX 15674 kb) [file 12864_2017_3641_MOESM5_ESM.pptx]

## Slide 1
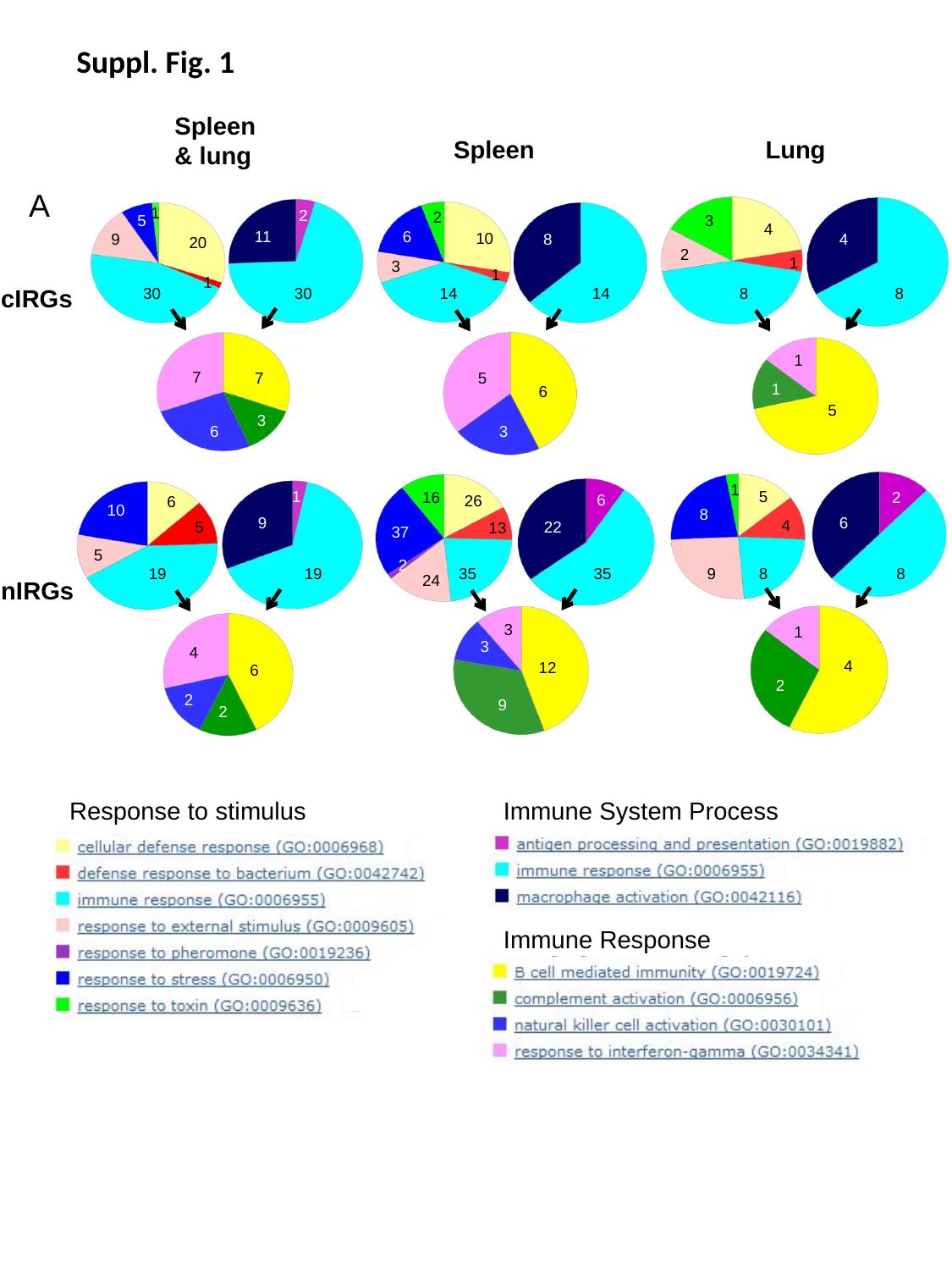

Suppl. Fig. 1
Spleen
& lung
Spleen
Lung
A
3
4
2
1
8
2
11
30
4
8
1
5
9
20
1
30
2
6
10
3
1
14
8
14
7
7
3
6
5
6
3
1
1
5
2
6
8
1
5
8
4
8
9
16
26
13
37
2
35
24
35
6
22
1
9
19
6
10
5
5
19
1
4
2
3
3
12
9
4
6
2
2
cIRGs
nIRGs
Response to stimulus
Immune System Process
Immune Response

## Slide 2
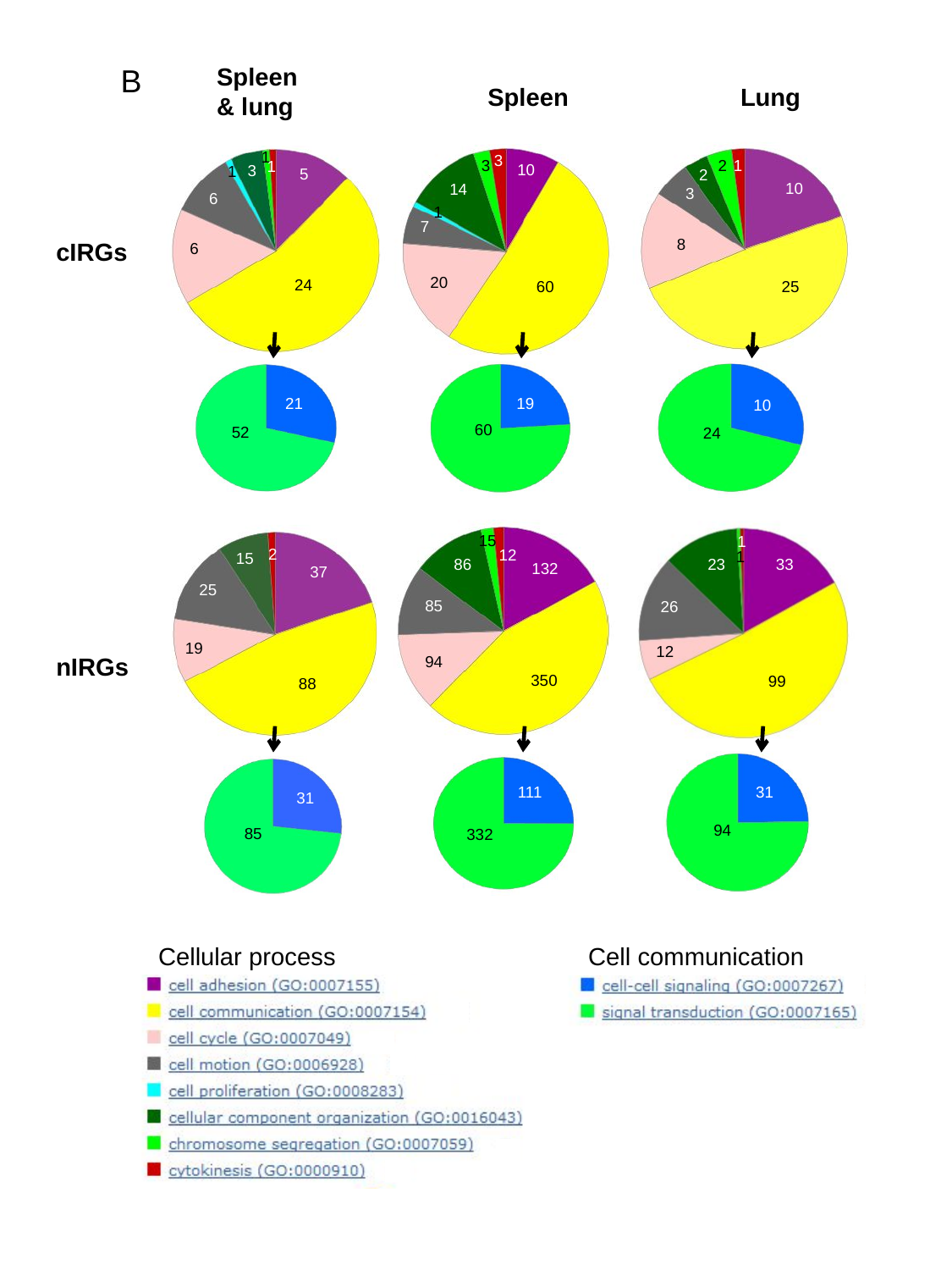

B
Spleen
& lung
Spleen
Lung
2
1
2
10
3
8
25
1
1
3
1
5
6
6
24
3
3
10
14
1
7
20
60
21
52
19
60
10
24
15
12
86
132
85
94
350
1
1
23
33
26
12
99
2
15
37
25
19
88
31
94
31
85
111
332
 cIRGs
 nIRGs
Cellular process
Cell communication
7

## Slide 3
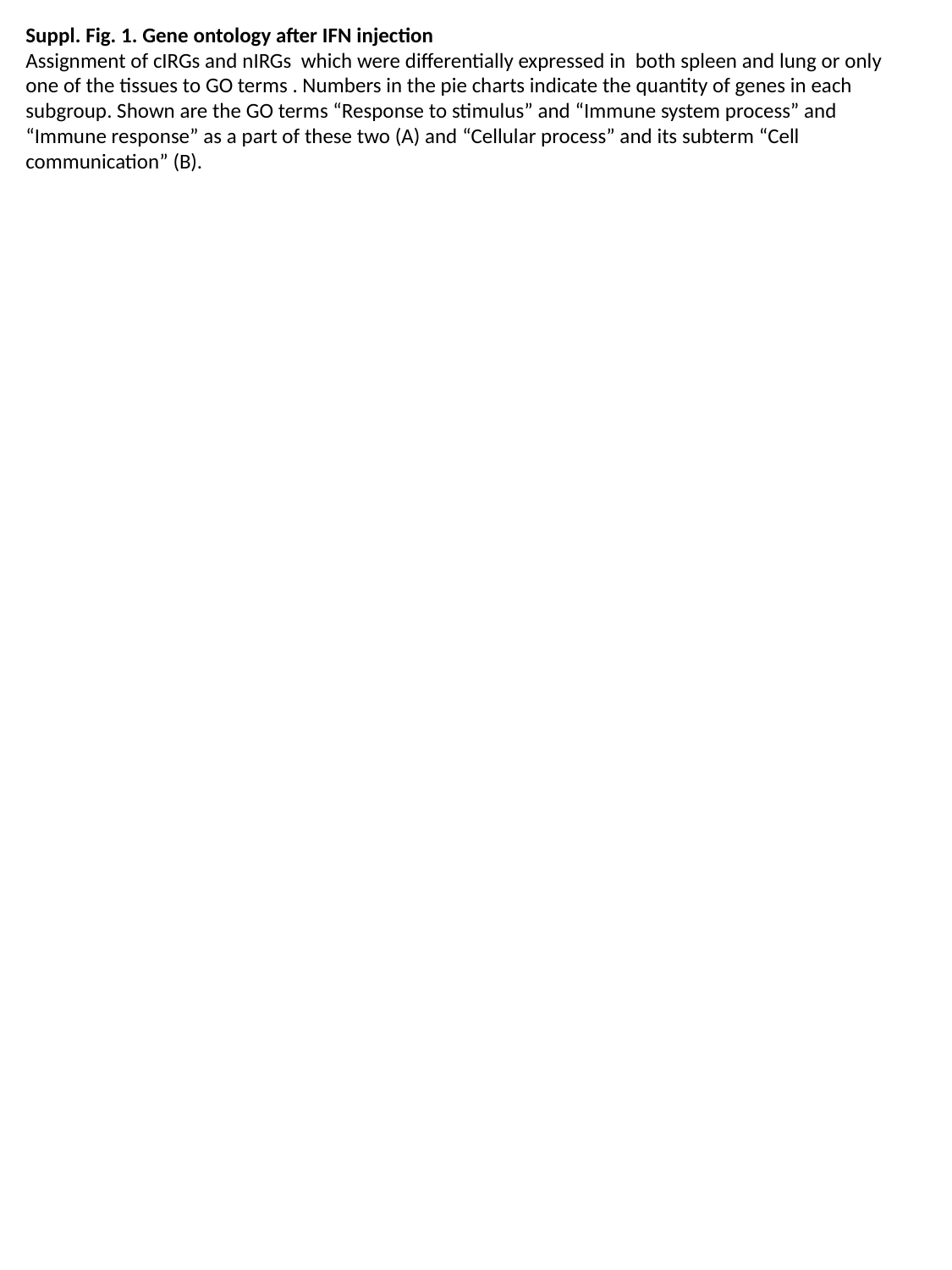

Suppl. Fig. 1. Gene ontology after IFN injection
Assignment of cIRGs and nIRGs which were differentially expressed in both spleen and lung or only one of the tissues to GO terms . Numbers in the pie charts indicate the quantity of genes in each subgroup. Shown are the GO terms “Response to stimulus” and “Immune system process” and “Immune response” as a part of these two (A) and “Cellular process” and its subterm “Cell communication” (B).
