# Supplementary figures and images for: Tissue and time specific expression pattern of interferon regulated genes in the chicken
Source: BMC Genomics. 2017 Mar 28;18:264. doi: 10.1186/s12864-017-3641-6 (PMC5371264; doi:10.1186/s12864-017-3641-6)

## Slide 1
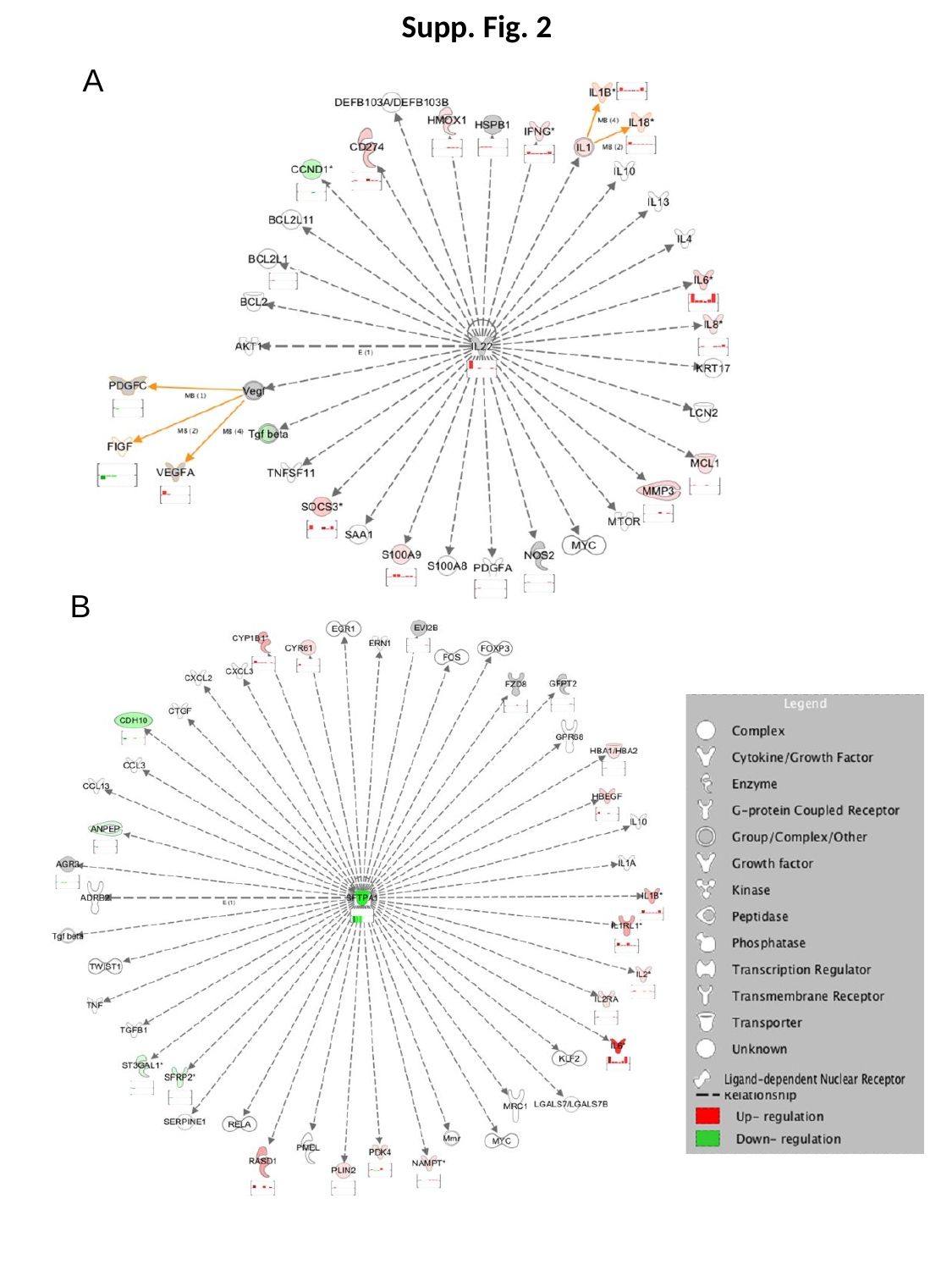

Supp. Fig. 2
A
B

Supplement: Supplementary file 6 — IPA network analysis for IL22 and SFTPA1. Gene interactions of IL22 (A) and SFTPA1 (B) obtained by IPA. Genes with higher mRNA abundance in the IFN treated animals are shown in red, genes with lower mRNA abundance in the treated animals in green. The small diagrams next to each differentially expressed gene display expression (FC) at the different time points. (PPTX 2050 kb) [file 12864_2017_3641_MOESM6_ESM.pptx]
